# Supplementary material for: PIEZO1 and PECAM1 interact at cell-cell junctions and partner in endothelial force sensing
Source: Commun Biol. 2023 Apr 1;6:358. doi: 10.1038/s42003-023-04706-4 (PMC10067937; doi:10.1038/s42003-023-04706-4)
Supplement: Supplementary file 2 — Supplementary Information-New [file 42003_2023_4706_MOESM2_ESM.pptx]

## Slide 1
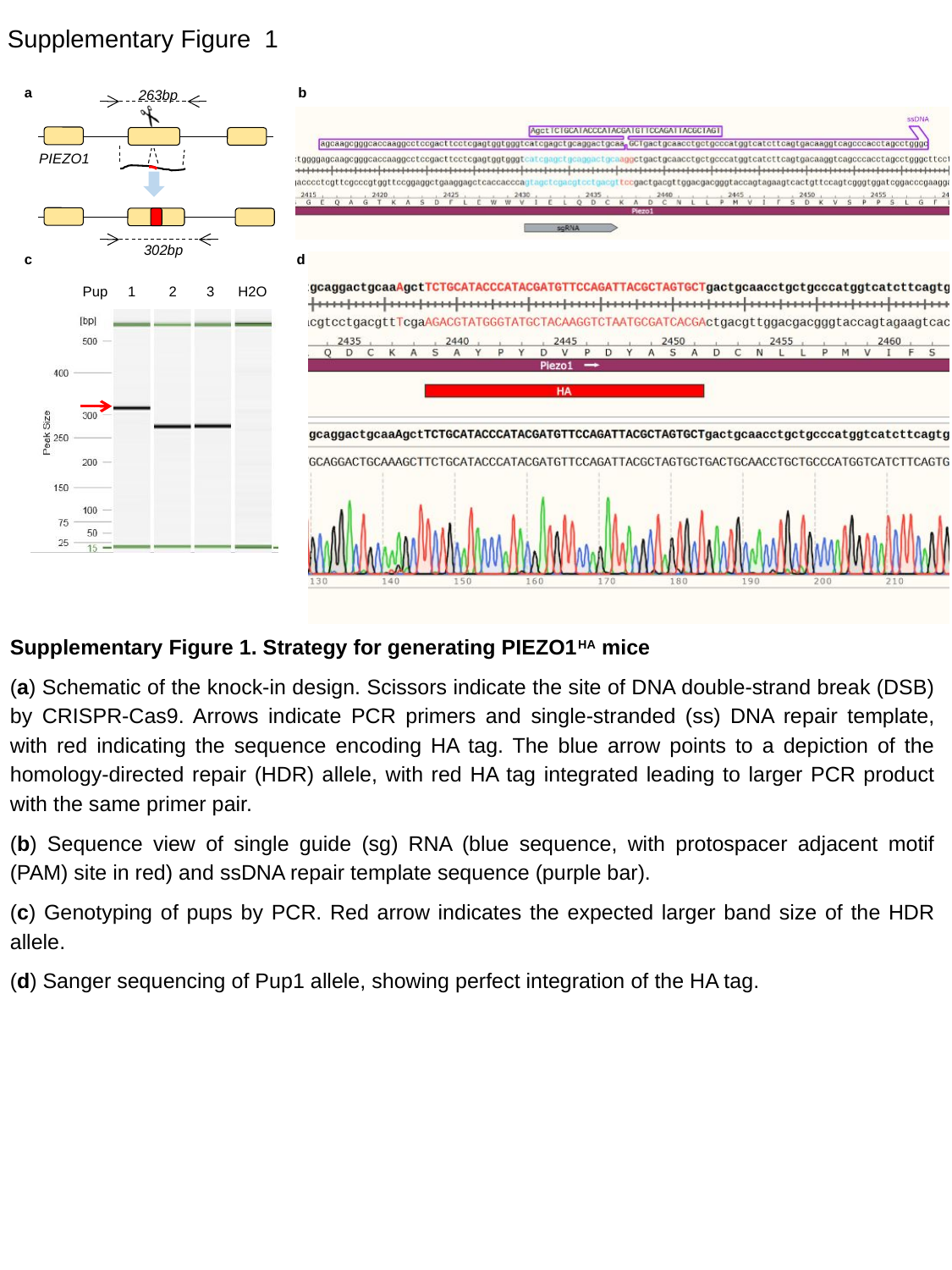

Supplementary Figure 1
a
b
263bp
PIEZO1
302bp
c
d
Pup
1
2
3
H2O
Supplementary Figure 1. Strategy for generating PIEZO1HA mice
(a) Schematic of the knock-in design. Scissors indicate the site of DNA double-strand break (DSB) by CRISPR-Cas9. Arrows indicate PCR primers and single-stranded (ss) DNA repair template, with red indicating the sequence encoding HA tag. The blue arrow points to a depiction of the homology-directed repair (HDR) allele, with red HA tag integrated leading to larger PCR product with the same primer pair.
(b) Sequence view of single guide (sg) RNA (blue sequence, with protospacer adjacent motif (PAM) site in red) and ssDNA repair template sequence (purple bar).
(c) Genotyping of pups by PCR. Red arrow indicates the expected larger band size of the HDR allele.
(d) Sanger sequencing of Pup1 allele, showing perfect integration of the HA tag.

## Slide 2
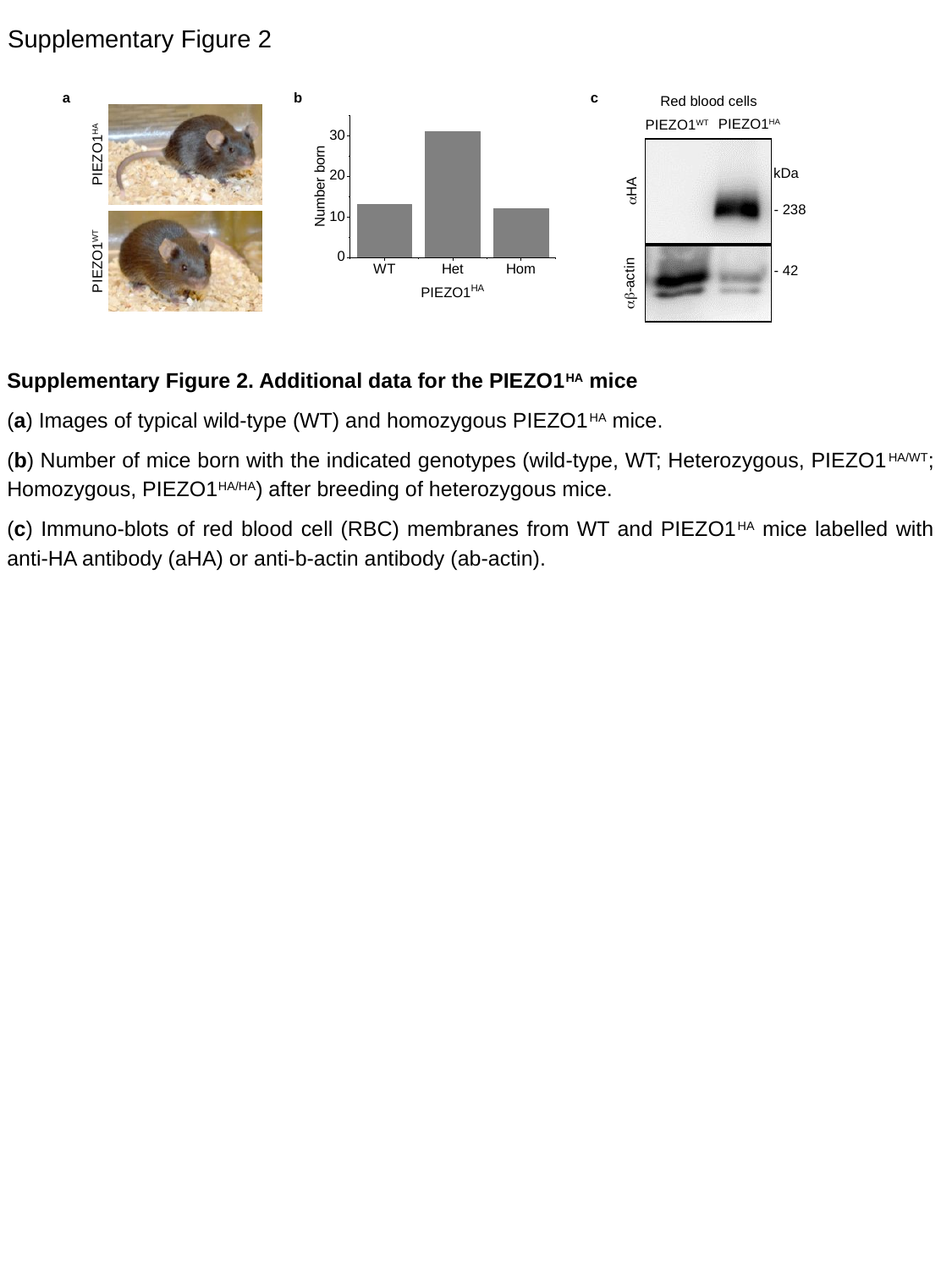

Supplementary Figure 2
a
b
c
Red blood cells
PIEZO1HA
PIEZO1WT
kDa
αHA
- 238
- 42
αβ-actin
PIEZO1HA
PIEZO1WT
Supplementary Figure 2. Additional data for the PIEZO1HA mice
(a) Images of typical wild-type (WT) and homozygous PIEZO1HA mice.
(b) Number of mice born with the indicated genotypes (wild-type, WT; Heterozygous, PIEZO1HA/WT; Homozygous, PIEZO1HA/HA) after breeding of heterozygous mice.
(c) Immuno-blots of red blood cell (RBC) membranes from WT and PIEZO1HA mice labelled with anti-HA antibody (aHA) or anti-b-actin antibody (ab-actin).

## Slide 3
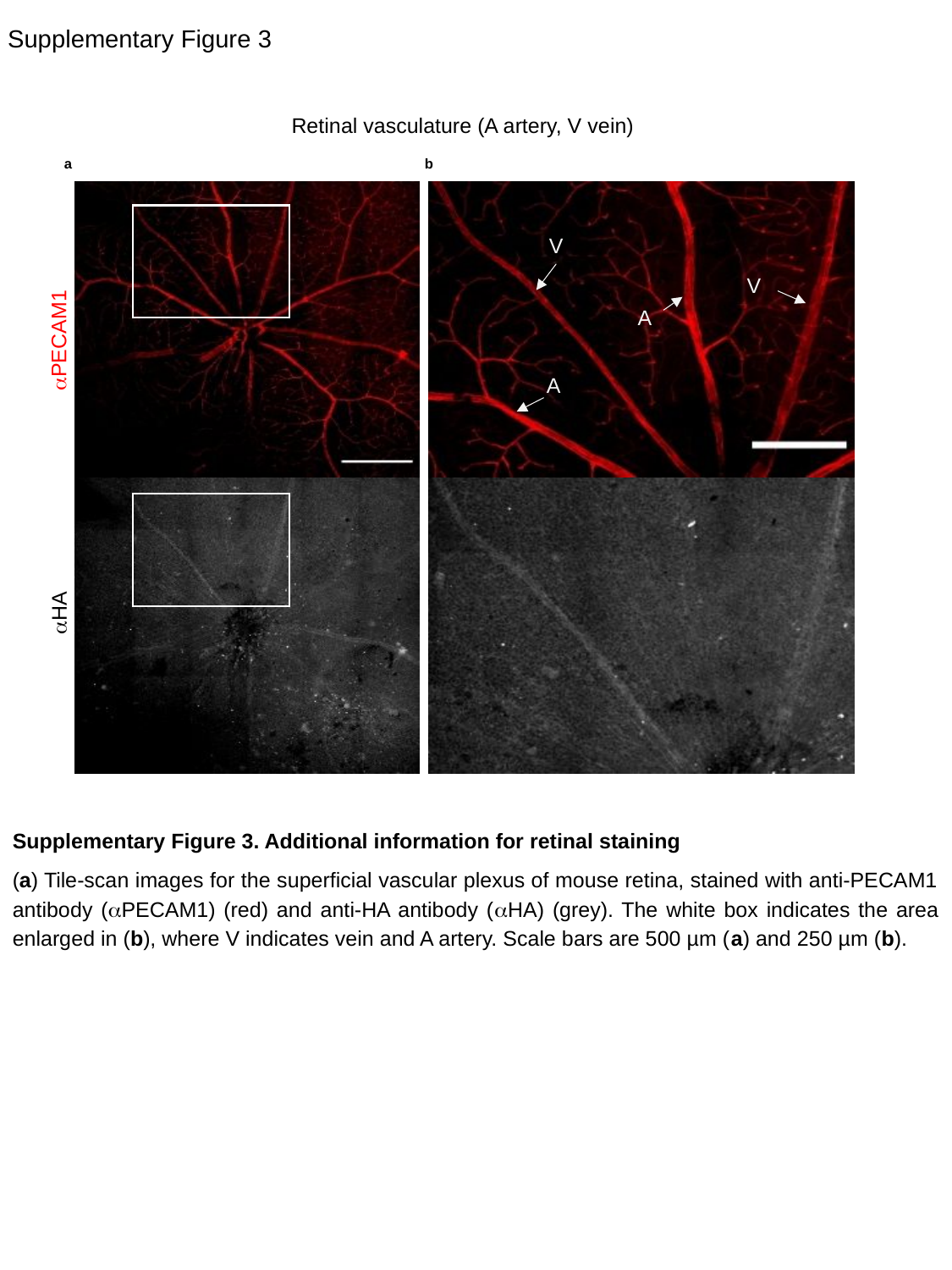

Supplementary Figure 3
Retinal vasculature (A artery, V vein)
a
b
V
V
A
A
αPECAM1
αHA
Supplementary Figure 3. Additional information for retinal staining
(a) Tile-scan images for the superficial vascular plexus of mouse retina, stained with anti-PECAM1 antibody (αPECAM1) (red) and anti-HA antibody (αHA) (grey). The white box indicates the area enlarged in (b), where V indicates vein and A artery. Scale bars are 500 µm (a) and 250 µm (b).

## Slide 4
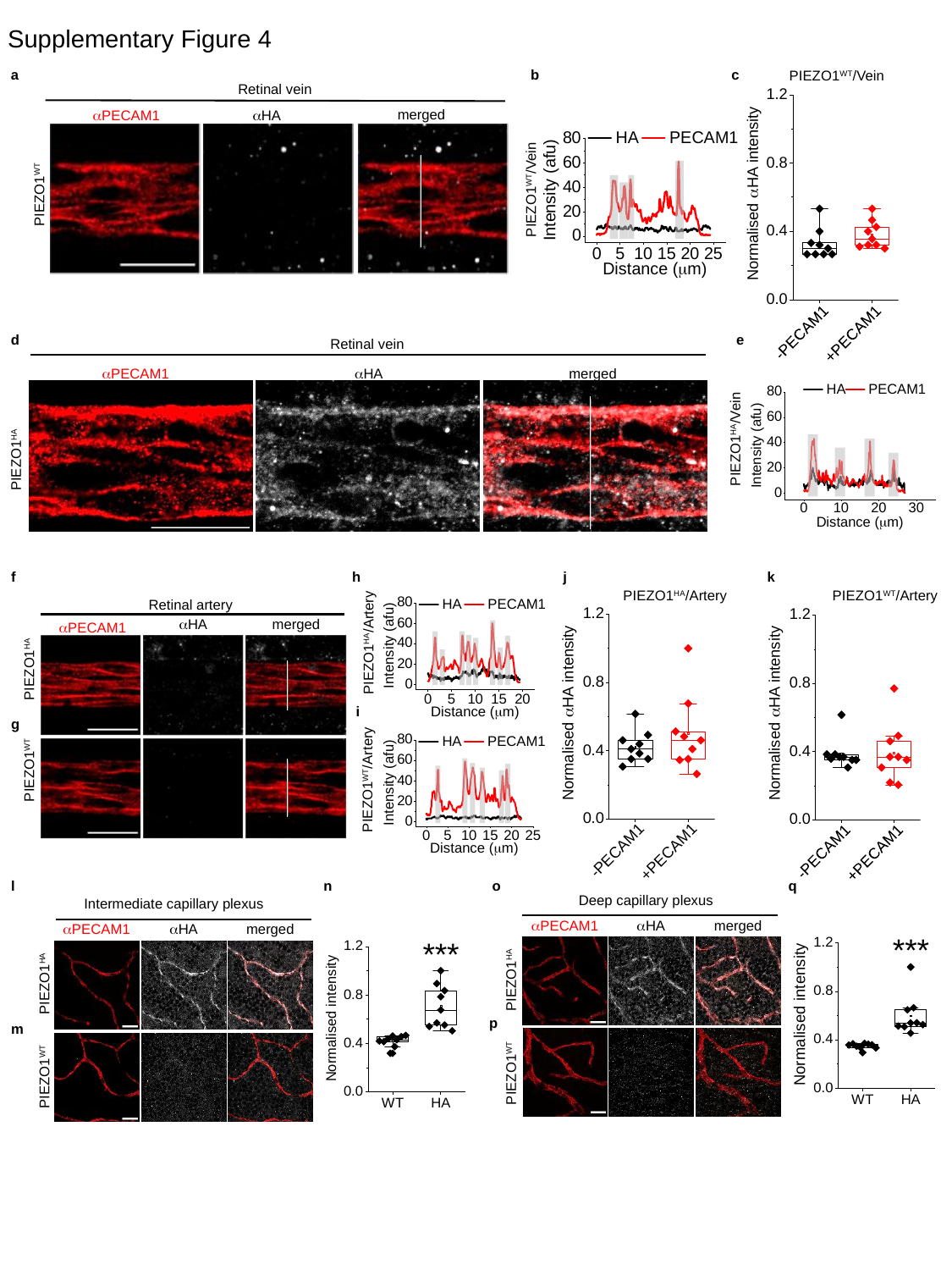

Supplementary Figure 4
a
b
c
PIEZO1WT/Vein
Retinal vein
merged
αPECAM1
αHA
PIEZO1WT/Vein
PIEZO1WT
d
e
Retinal vein
αPECAM1
αHA
merged
PIEZO1HA/Vein
PIEZO1HA
f
h
j
k
Retinal artery
αHA
merged
αPECAM1
PIEZO1HA/Artery
PIEZO1HA
PIEZO1WT
PIEZO1WT/Artery
PIEZO1HA/Artery
PIEZO1WT/Artery
i
g
l
n
o
q
Deep capillary plexus
αPECAM1
αHA
merged
PIEZO1HA
PIEZO1WT
Intermediate capillary plexus
αPECAM1
αHA
merged
PIEZO1HA
PIEZO1WT
p
m

## Slide 5
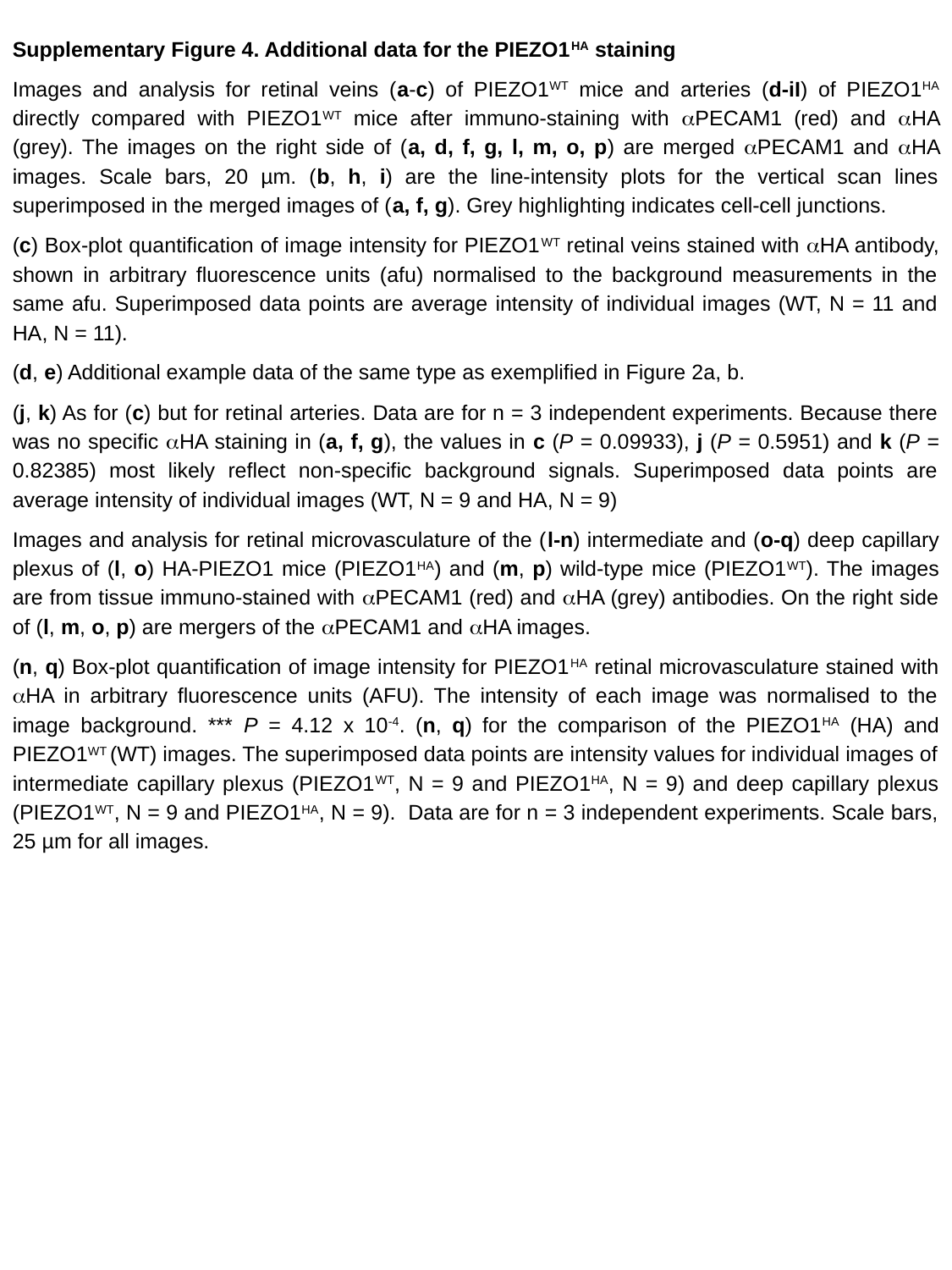

Supplementary Figure 4. Additional data for the PIEZO1HA staining
Images and analysis for retinal veins (a-c) of PIEZO1WT mice and arteries (d-iI) of PIEZO1HA directly compared with PIEZO1WT mice after immuno-staining with αPECAM1 (red) and αHA (grey). The images on the right side of (a, d, f, g, l, m, o, p) are merged αPECAM1 and αHA images. Scale bars, 20 µm. (b, h, i) are the line-intensity plots for the vertical scan lines superimposed in the merged images of (a, f, g). Grey highlighting indicates cell-cell junctions.
(c) Box-plot quantification of image intensity for PIEZO1WT retinal veins stained with αHA antibody, shown in arbitrary fluorescence units (afu) normalised to the background measurements in the same afu. Superimposed data points are average intensity of individual images (WT, N = 11 and HA, N = 11).
(d, e) Additional example data of the same type as exemplified in Figure 2a, b.
(j, k) As for (c) but for retinal arteries. Data are for n = 3 independent experiments. Because there was no specific αHA staining in (a, f, g), the values in c (P = 0.09933), j (P = 0.5951) and k (P = 0.82385) most likely reflect non-specific background signals. Superimposed data points are average intensity of individual images (WT, N = 9 and HA, N = 9)
Images and analysis for retinal microvasculature of the (l-n) intermediate and (o-q) deep capillary plexus of (l, o) HA-PIEZO1 mice (PIEZO1HA) and (m, p) wild-type mice (PIEZO1WT). The images are from tissue immuno-stained with αPECAM1 (red) and αHA (grey) antibodies. On the right side of (l, m, o, p) are mergers of the αPECAM1 and αHA images.
(n, q) Box-plot quantification of image intensity for PIEZO1HA retinal microvasculature stained with αHA in arbitrary fluorescence units (AFU). The intensity of each image was normalised to the image background. *** P = 4.12 x 10^-4^. (n, q) for the comparison of the PIEZO1HA (HA) and PIEZO1WT (WT) images. The superimposed data points are intensity values for individual images of intermediate capillary plexus (PIEZO1WT, N = 9 and PIEZO1HA, N = 9) and deep capillary plexus (PIEZO1WT, N = 9 and PIEZO1HA, N = 9). Data are for n = 3 independent experiments. Scale bars, 25 µm for all images.

## Slide 6
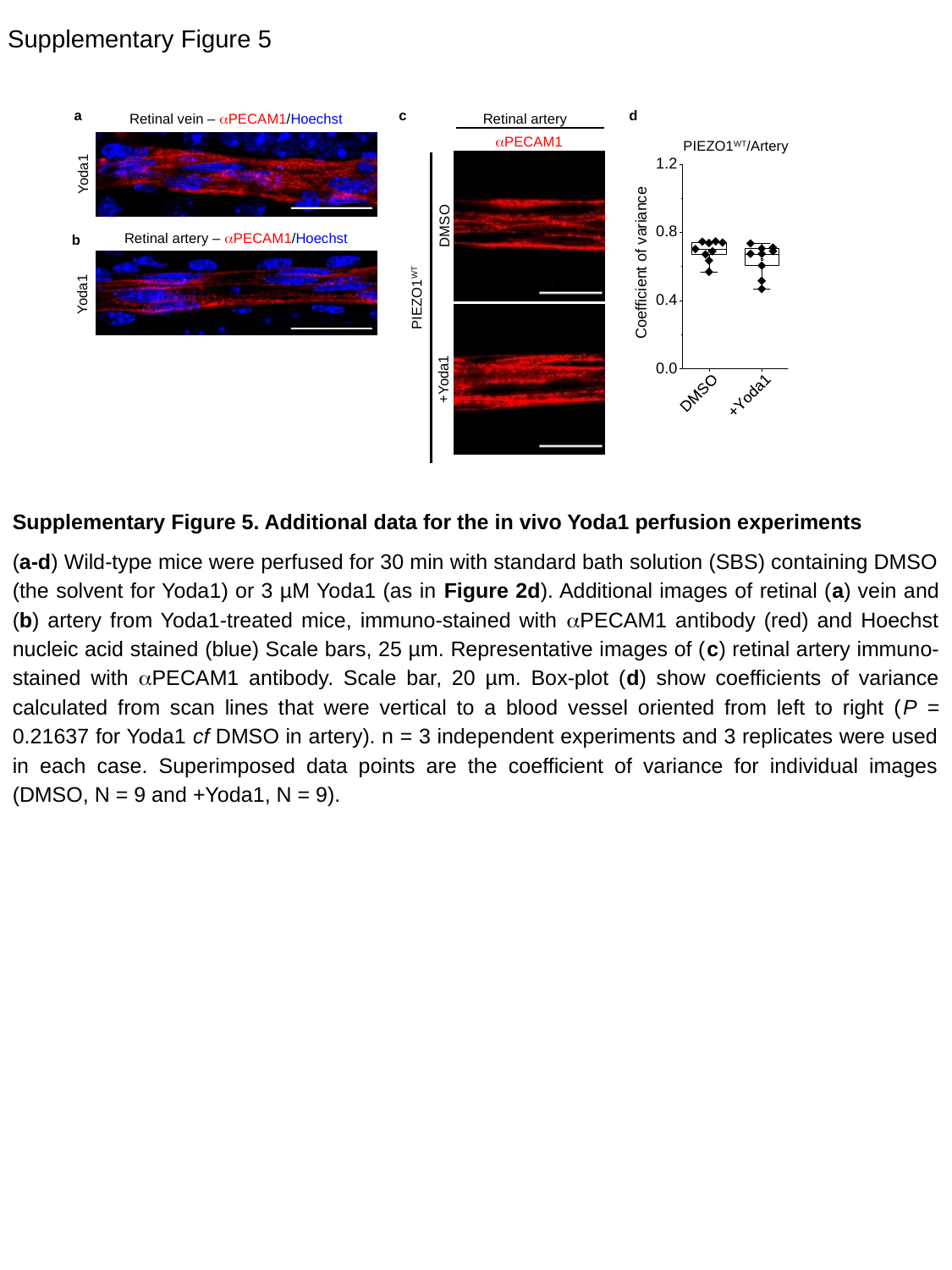

Supplementary Figure 5
a
c
d
Retinal vein – αPECAM1/Hoechst
Retinal artery
αPECAM1
PIEZO1WT/Artery
Yoda1
DMSO
Retinal artery – αPECAM1/Hoechst
b
Yoda1
PIEZO1WT
+Yoda1
Supplementary Figure 5. Additional data for the in vivo Yoda1 perfusion experiments
(a-d) Wild-type mice were perfused for 30 min with standard bath solution (SBS) containing DMSO (the solvent for Yoda1) or 3 µM Yoda1 (as in Figure 2d). Additional images of retinal (a) vein and (b) artery from Yoda1-treated mice, immuno-stained with αPECAM1 antibody (red) and Hoechst nucleic acid stained (blue) Scale bars, 25 µm. Representative images of (c) retinal artery immuno-stained with αPECAM1 antibody. Scale bar, 20 µm. Box-plot (d) show coefficients of variance calculated from scan lines that were vertical to a blood vessel oriented from left to right (P = 0.21637 for Yoda1 cf DMSO in artery). n = 3 independent experiments and 3 replicates were used in each case. Superimposed data points are the coefficient of variance for individual images (DMSO, N = 9 and +Yoda1, N = 9).

## Slide 7
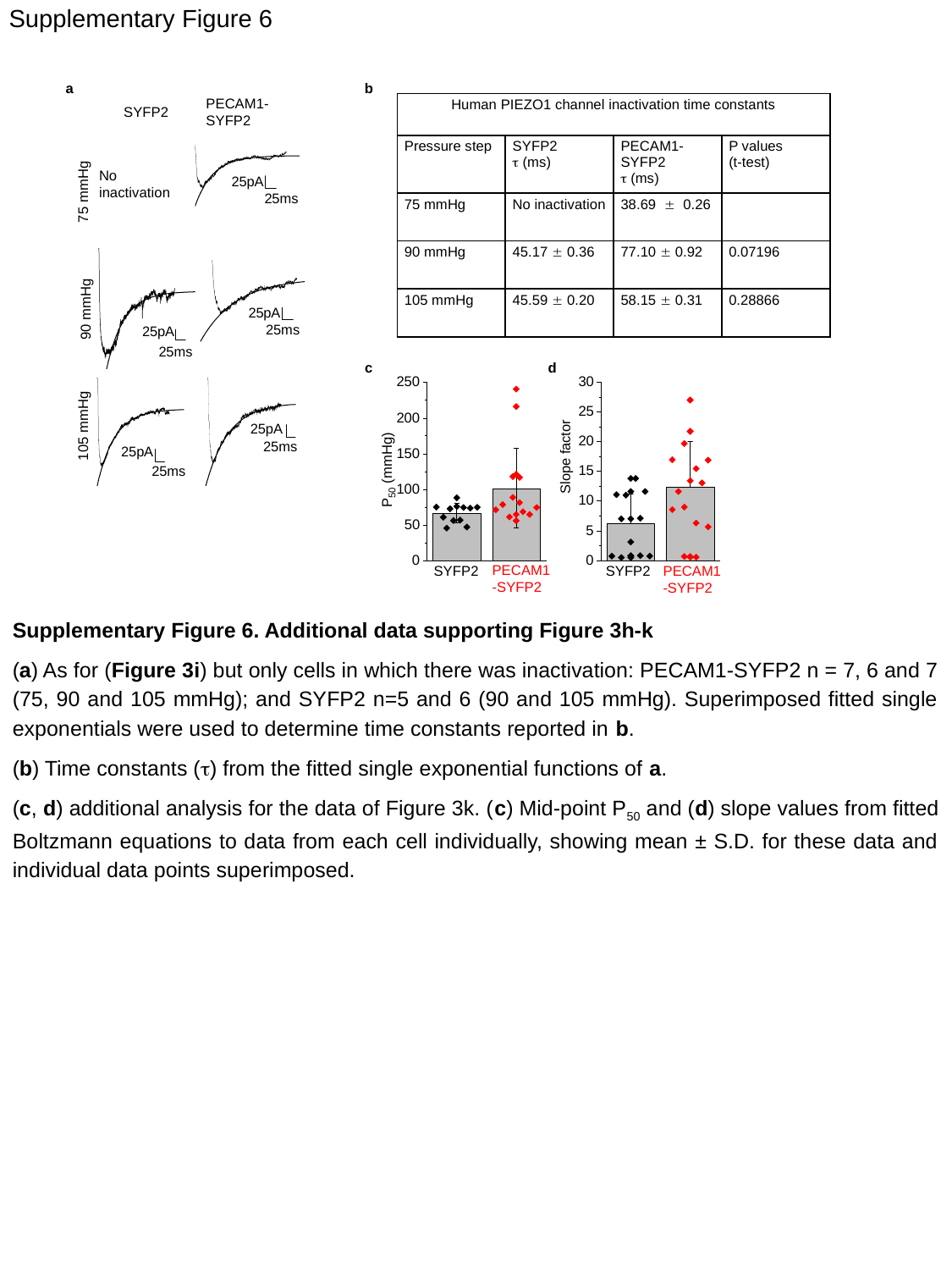

Supplementary Figure 6
PECAM1-SYFP2
a
b
SYFP2
| Human PIEZO1 channel inactivation time constants | | | |
| --- | --- | --- | --- |
| Pressure step | SYFP2  (ms) | PECAM1-SYFP2  (ms) | P values (t-test) |
| 75 mmHg | No inactivation | 38.69 ± 0.26 | |
| 90 mmHg | 45.17 ± 0.36 | 77.10 ± 0.92 | 0.07196 |
| 105 mmHg | 45.59 ± 0.20 | 58.15 ± 0.31 | 0.28866 |
No inactivation
75 mmHg
90 mmHg
c
d
105 mmHg
Supplementary Figure 6. Additional data supporting Figure 3h-k
(a) As for (Figure 3i) but only cells in which there was inactivation: PECAM1-SYFP2 n = 7, 6 and 7 (75, 90 and 105 mmHg); and SYFP2 n=5 and 6 (90 and 105 mmHg). Superimposed fitted single exponentials were used to determine time constants reported in b.
(b) Time constants () from the fitted single exponential functions of a.
(c, d) additional analysis for the data of Figure 3k. (c) Mid-point P50 and (d) slope values from fitted Boltzmann equations to data from each cell individually, showing mean ± S.D. for these data and individual data points superimposed.

## Slide 8
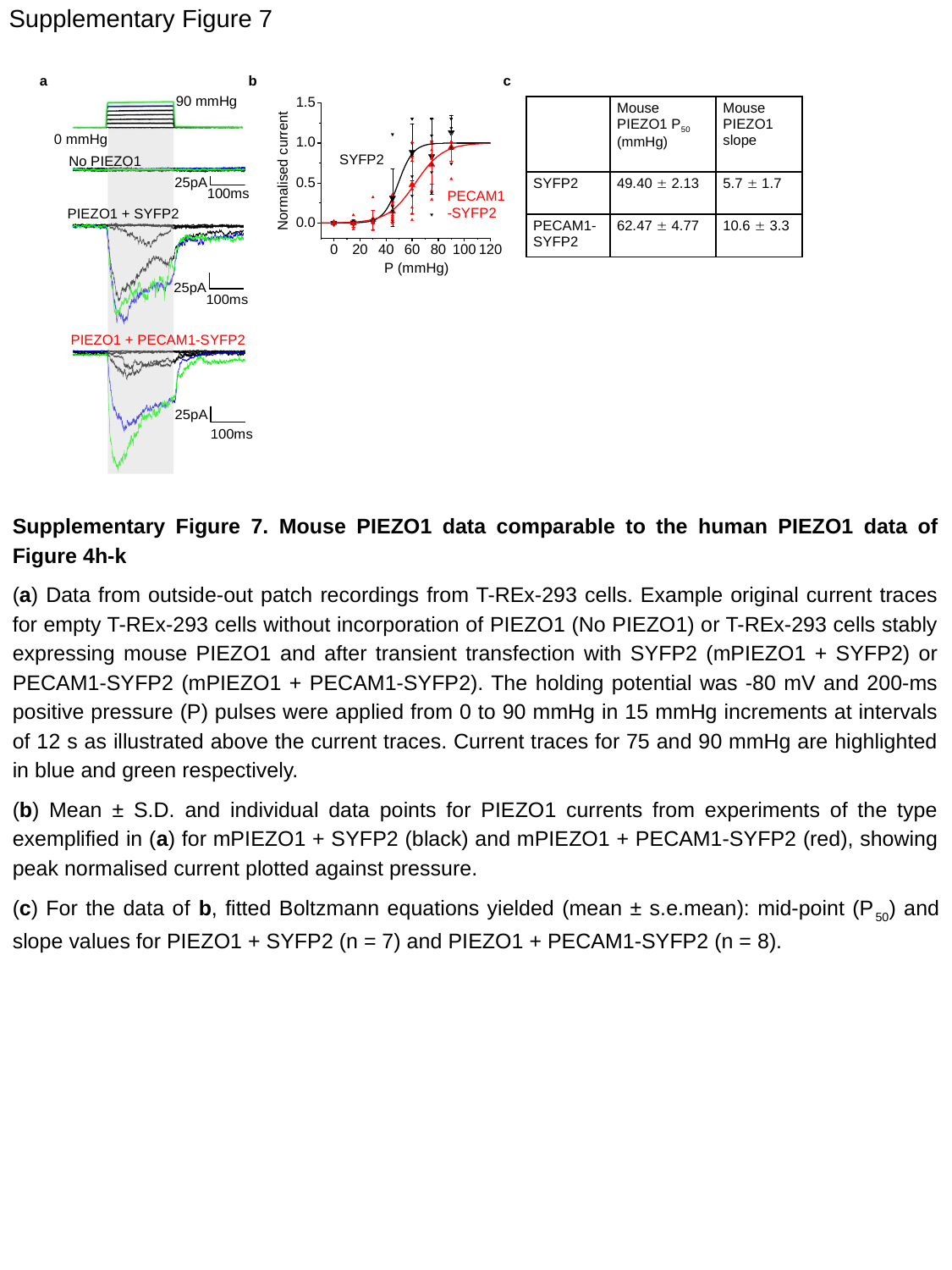

Supplementary Figure 7
a
b
c
90 mmHg
No PIEZO1
PIEZO1 + SYFP2
PIEZO1 + PECAM1-SYFP2
| | Mouse PIEZO1 P50 (mmHg) | Mouse PIEZO1 slope |
| --- | --- | --- |
| SYFP2 | 49.40 ± 2.13 | 5.7 ± 1.7 |
| PECAM1- SYFP2 | 62.47 ± 4.77 | 10.6 ± 3.3 |
0 mmHg
Supplementary Figure 7. Mouse PIEZO1 data comparable to the human PIEZO1 data of Figure 4h-k
(a) Data from outside-out patch recordings from T-REx-293 cells. Example original current traces for empty T-REx-293 cells without incorporation of PIEZO1 (No PIEZO1) or T-REx-293 cells stably expressing mouse PIEZO1 and after transient transfection with SYFP2 (mPIEZO1 + SYFP2) or PECAM1-SYFP2 (mPIEZO1 + PECAM1-SYFP2). The holding potential was -80 mV and 200-ms positive pressure (P) pulses were applied from 0 to 90 mmHg in 15 mmHg increments at intervals of 12 s as illustrated above the current traces. Current traces for 75 and 90 mmHg are highlighted in blue and green respectively.
(b) Mean ± S.D. and individual data points for PIEZO1 currents from experiments of the type exemplified in (a) for mPIEZO1 + SYFP2 (black) and mPIEZO1 + PECAM1-SYFP2 (red), showing peak normalised current plotted against pressure.
(c) For the data of b, fitted Boltzmann equations yielded (mean ± s.e.mean): mid-point (P50) and slope values for PIEZO1 + SYFP2 (n = 7) and PIEZO1 + PECAM1-SYFP2 (n = 8).

## Slide 9
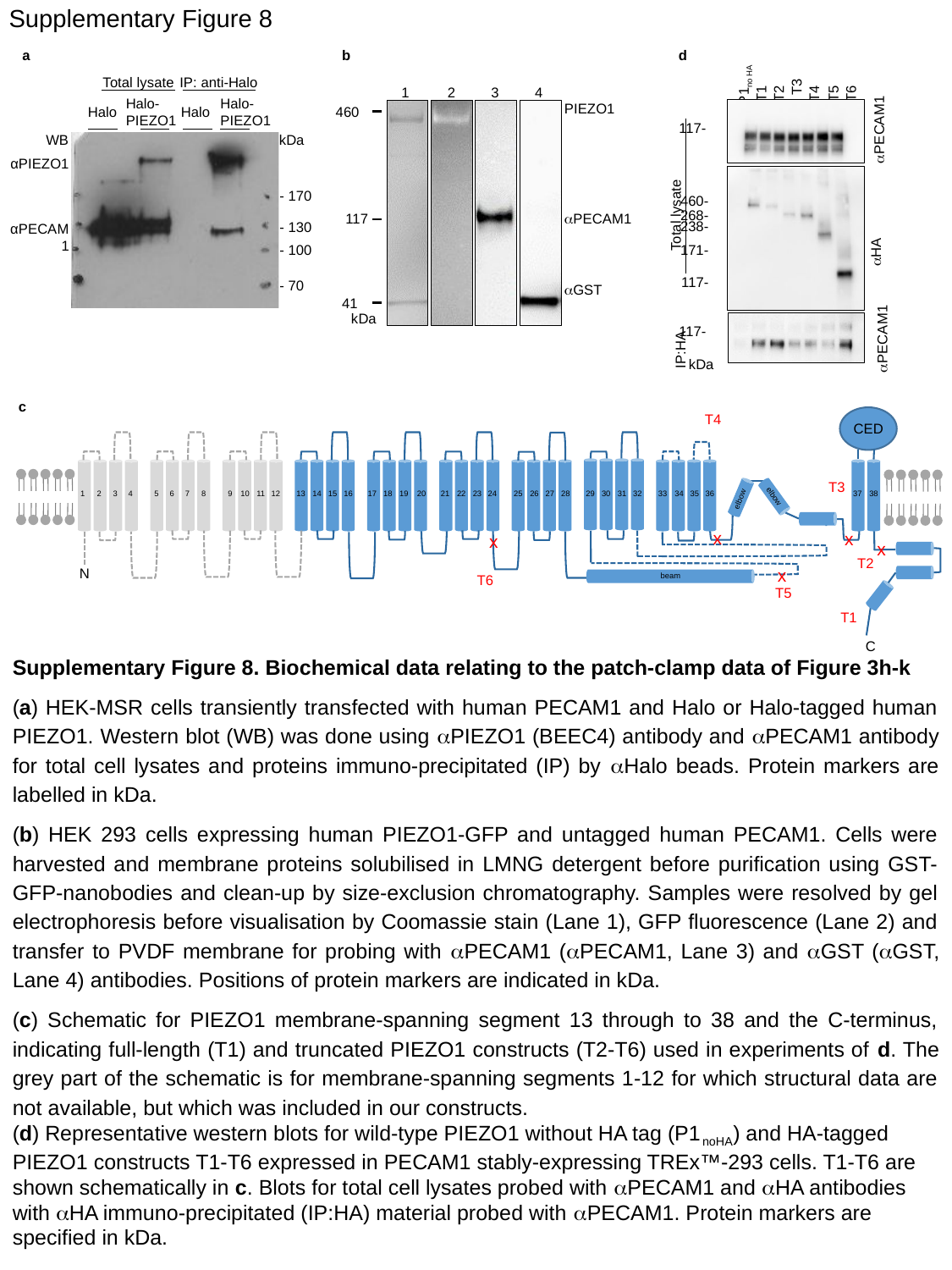

Supplementary Figure 8
a
b
d
T3
P1no HA
T1
T2
T4
T5
T6
117-
αPECAM1
460-
268-
238-
171-
117-
Total lysate
αHA
117-
αPECAM1
IP:HA
kDa
IP: anti-Halo
Total lysate
Halo-
PIEZO1
Halo-
PIEZO1
Halo
Halo
WB
kDa
αPIEZO1
- 170
- 130
αPECAM1
- 100
- 70
1
2
3
4
PIEZO1
460
117
αPECAM1
αGST
41
kDa
c
T4
1
2
3
4
5
6
7
8
9
10
11
12
13
14
15
16
17
18
19
20
21
22
23
24
25
26
27
28
29
30
31
32
33
34
35
36
37
38
CED
elbow
elbow
N
beam
C
T3
x
x
x
x
T2
x
T6
T5
T1
Supplementary Figure 8. Biochemical data relating to the patch-clamp data of Figure 3h-k
(a) HEK-MSR cells transiently transfected with human PECAM1 and Halo or Halo-tagged human PIEZO1. Western blot (WB) was done using αPIEZO1 (BEEC4) antibody and αPECAM1 antibody for total cell lysates and proteins immuno-precipitated (IP) by αHalo beads. Protein markers are labelled in kDa.
(b) HEK 293 cells expressing human PIEZO1-GFP and untagged human PECAM1. Cells were harvested and membrane proteins solubilised in LMNG detergent before purification using GST-GFP-nanobodies and clean-up by size-exclusion chromatography. Samples were resolved by gel electrophoresis before visualisation by Coomassie stain (Lane 1), GFP fluorescence (Lane 2) and transfer to PVDF membrane for probing with αPECAM1 (aPECAM1, Lane 3) and αGST (aGST, Lane 4) antibodies. Positions of protein markers are indicated in kDa.
(c) Schematic for PIEZO1 membrane-spanning segment 13 through to 38 and the C-terminus, indicating full-length (T1) and truncated PIEZO1 constructs (T2-T6) used in experiments of d. The grey part of the schematic is for membrane-spanning segments 1-12 for which structural data are not available, but which was included in our constructs.
(d) Representative western blots for wild-type PIEZO1 without HA tag (P1noHA) and HA-tagged PIEZO1 constructs T1-T6 expressed in PECAM1 stably-expressing TREx™-293 cells. T1-T6 are shown schematically in c. Blots for total cell lysates probed with αPECAM1 and αHA antibodies with αHA immuno-precipitated (IP:HA) material probed with aPECAM1. Protein markers are specified in kDa.

## Slide 10
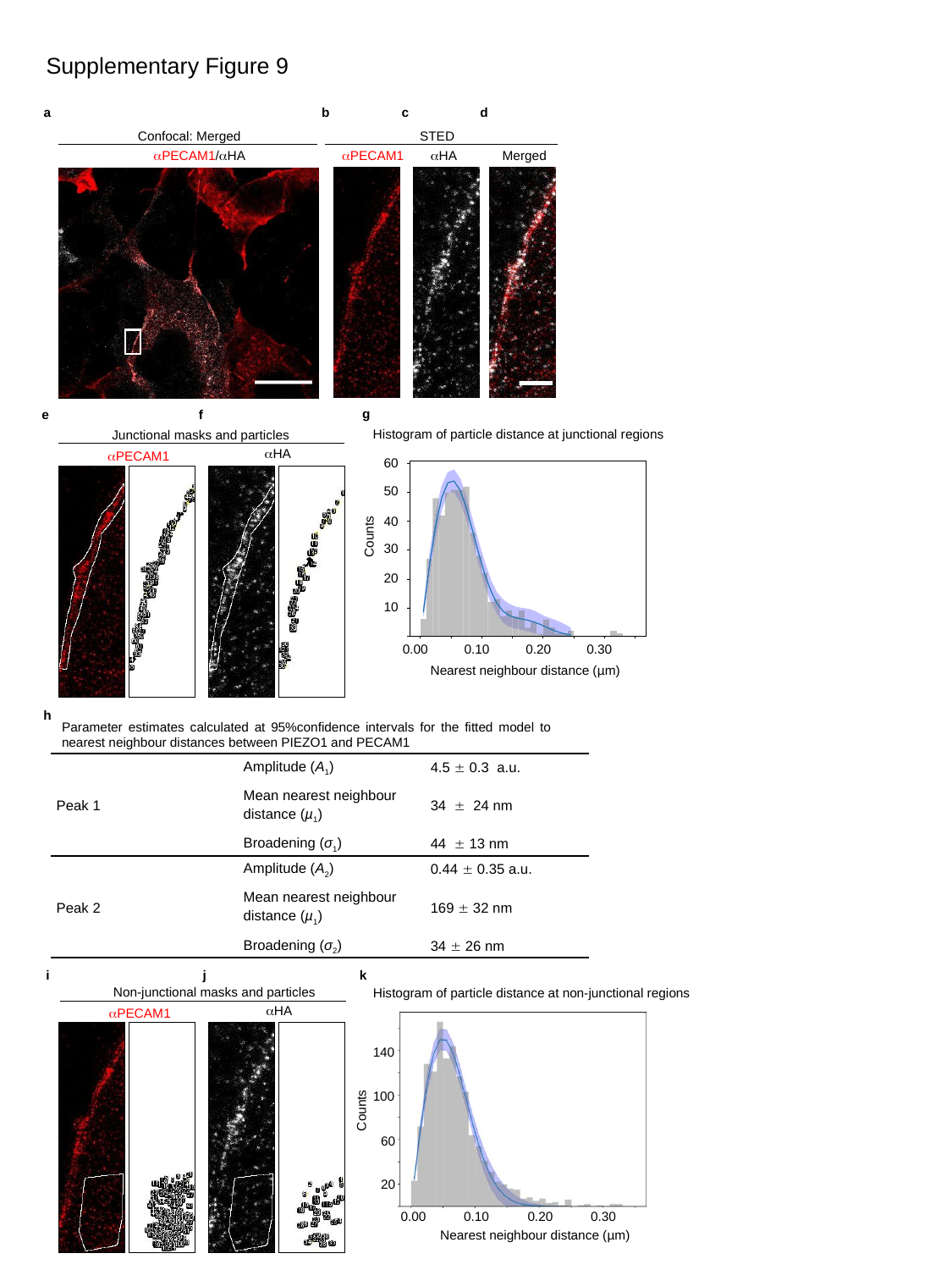

Supplementary Figure 9
a
b
c
d
Confocal: Merged
STED
αPECAM1/αHA
αPECAM1
αHA
Merged
g
e
f
Histogram of particle distance at junctional regions
Junctional masks and particles
αHA
αPECAM1
60
50
40
30
20
10
Counts
0.10
0.00
0.20
0.30
Nearest neighbour distance (µm)
h
Parameter estimates calculated at 95%confidence intervals for the fitted model to nearest neighbour distances between PIEZO1 and PECAM1
| Peak 1 | Amplitude (A1) | 4.5 ± 0.3 a.u. |
| --- | --- | --- |
| | Mean nearest neighbour distance (µ1) | 34 ± 24 nm |
| | Broadening (σ1) | 44 ± 13 nm |
| Peak 2 | Amplitude (A2) | 0.44 ± 0.35 a.u. |
| | Mean nearest neighbour distance (µ1) | 169 ± 32 nm |
| | Broadening (σ2) | 34 ± 26 nm |
i
j
k
Non-junctional masks and particles
αHA
αPECAM1
Histogram of particle distance at non-junctional regions
140
100
Counts
60
20
0.00
0.10
0.20
0.30
Nearest neighbour distance (µm)

## Slide 11
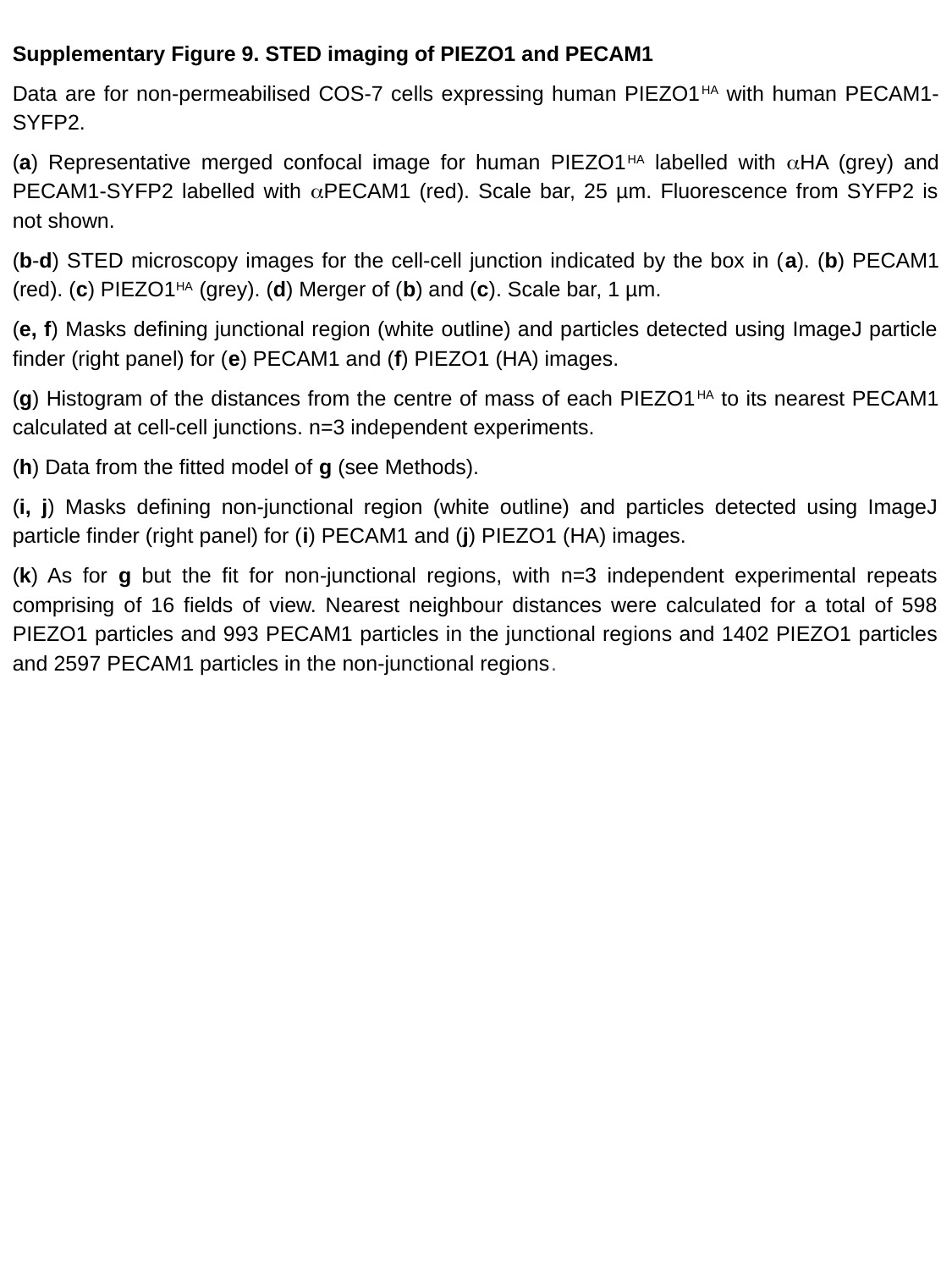

Supplementary Figure 9. STED imaging of PIEZO1 and PECAM1
Data are for non-permeabilised COS-7 cells expressing human PIEZO1HA with human PECAM1-SYFP2.
(a) Representative merged confocal image for human PIEZO1HA labelled with αHA (grey) and PECAM1-SYFP2 labelled with αPECAM1 (red). Scale bar, 25 µm. Fluorescence from SYFP2 is not shown.
(b-d) STED microscopy images for the cell-cell junction indicated by the box in (a). (b) PECAM1 (red). (c) PIEZO1HA (grey). (d) Merger of (b) and (c). Scale bar, 1 µm.
(e, f) Masks defining junctional region (white outline) and particles detected using ImageJ particle finder (right panel) for (e) PECAM1 and (f) PIEZO1 (HA) images.
(g) Histogram of the distances from the centre of mass of each PIEZO1HA to its nearest PECAM1 calculated at cell-cell junctions. n=3 independent experiments.
(h) Data from the fitted model of g (see Methods).
(i, j) Masks defining non-junctional region (white outline) and particles detected using ImageJ particle finder (right panel) for (i) PECAM1 and (j) PIEZO1 (HA) images.
(k) As for g but the fit for non-junctional regions, with n=3 independent experimental repeats comprising of 16 fields of view. Nearest neighbour distances were calculated for a total of 598 PIEZO1 particles and 993 PECAM1 particles in the junctional regions and 1402 PIEZO1 particles and 2597 PECAM1 particles in the non-junctional regions.

## Slide 12
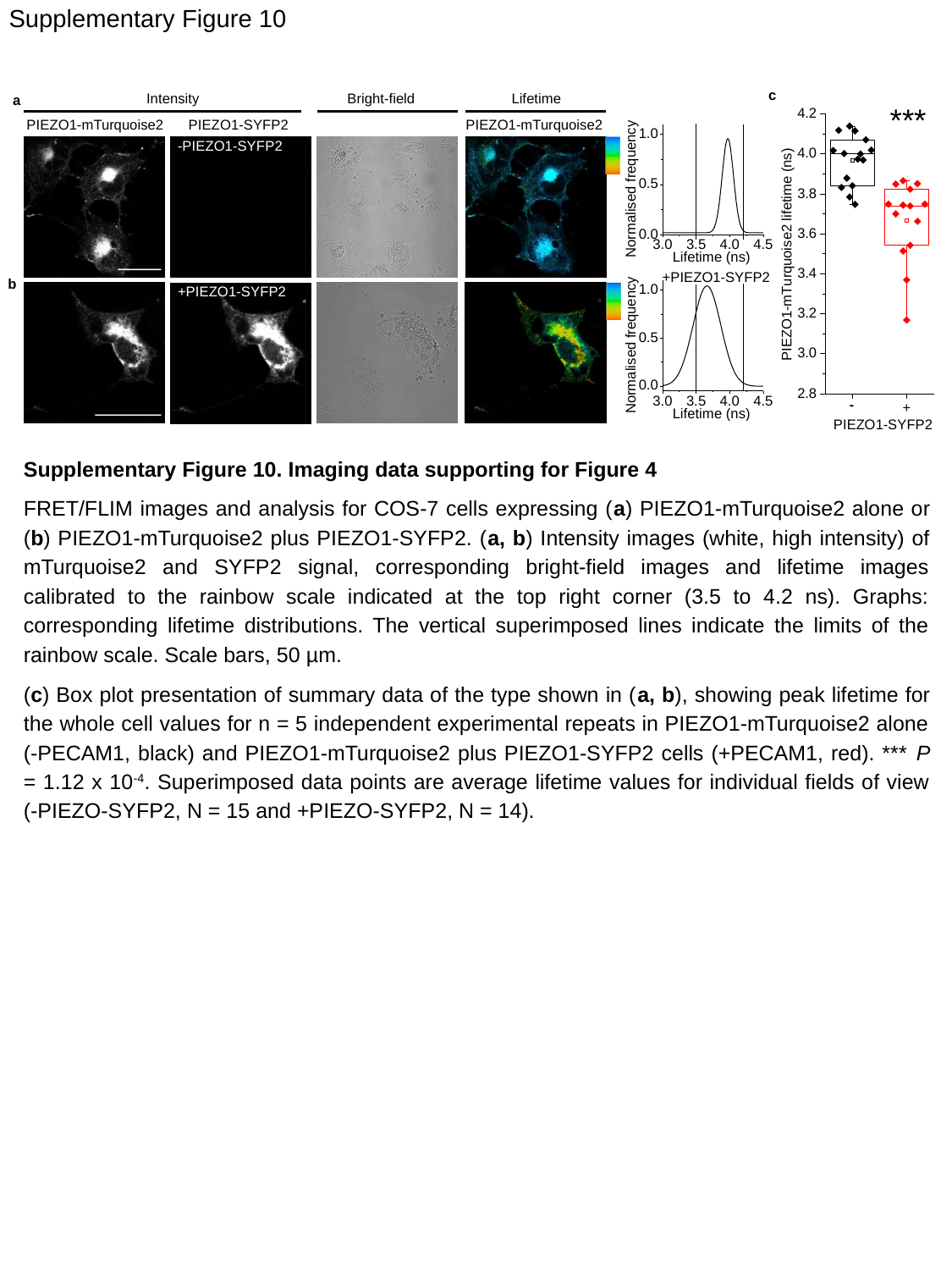

Supplementary Figure 10
c
Intensity
Bright-field
Lifetime
PIEZO1-mTurquoise2
PIEZO1-SYFP2
PIEZO1-mTurquoise2
-PIEZO1-SYFP2
+PIEZO1-SYFP2
a
b
Supplementary Figure 10. Imaging data supporting for Figure 4
FRET/FLIM images and analysis for COS-7 cells expressing (a) PIEZO1-mTurquoise2 alone or (b) PIEZO1-mTurquoise2 plus PIEZO1-SYFP2. (a, b) Intensity images (white, high intensity) of mTurquoise2 and SYFP2 signal, corresponding bright-field images and lifetime images calibrated to the rainbow scale indicated at the top right corner (3.5 to 4.2 ns). Graphs: corresponding lifetime distributions. The vertical superimposed lines indicate the limits of the rainbow scale. Scale bars, 50 µm.
(c) Box plot presentation of summary data of the type shown in (a, b), showing peak lifetime for the whole cell values for n = 5 independent experimental repeats in PIEZO1-mTurquoise2 alone (-PECAM1, black) and PIEZO1-mTurquoise2 plus PIEZO1-SYFP2 cells (+PECAM1, red). *** P = 1.12 x 10^-4^. Superimposed data points are average lifetime values for individual fields of view (-PIEZO-SYFP2, N = 15 and +PIEZO-SYFP2, N = 14).

## Slide 13
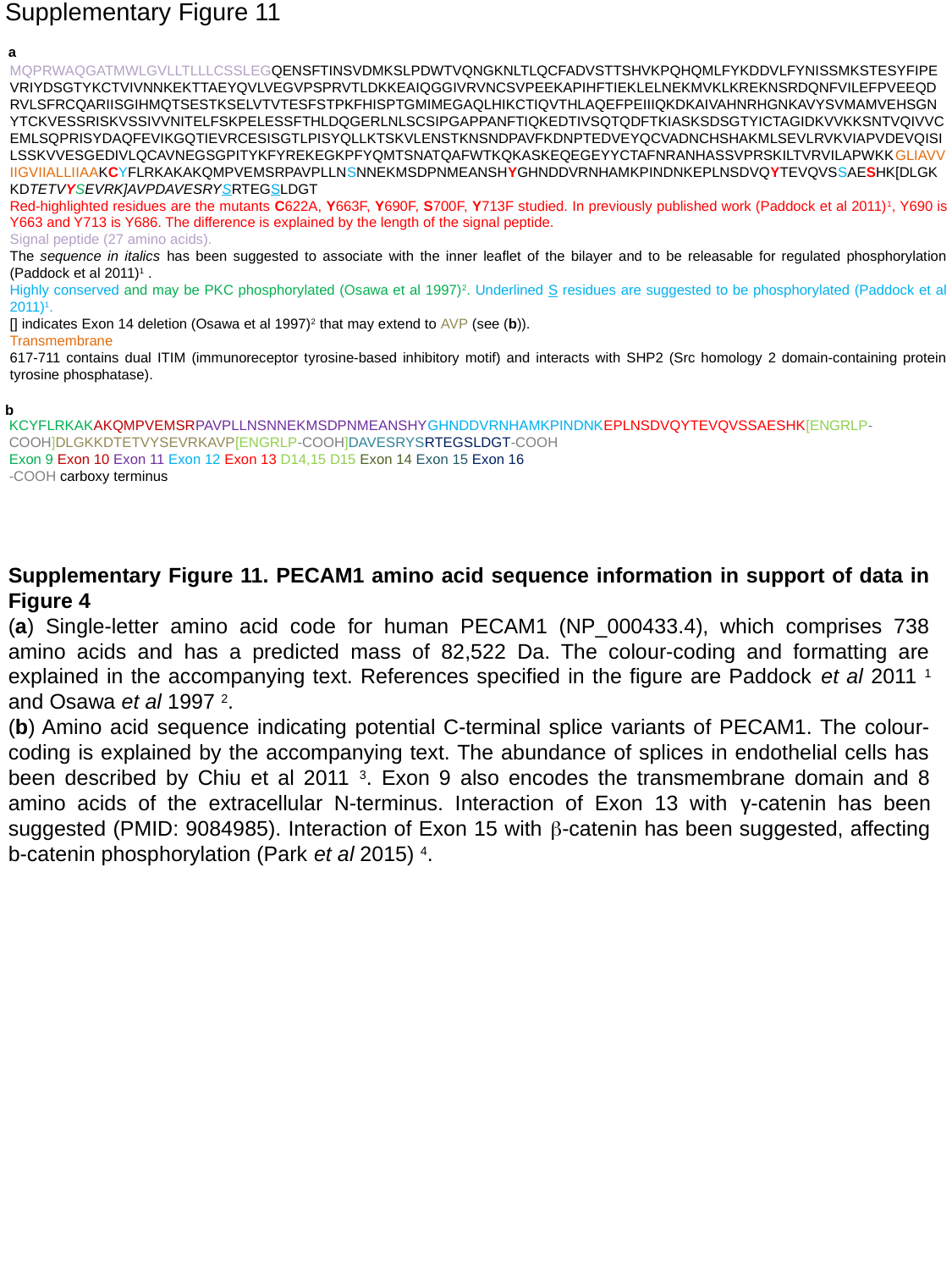

Supplementary Figure 11
a
MQPRWAQGATMWLGVLLTLLLCSSLEGQENSFTINSVDMKSLPDWTVQNGKNLTLQCFADVSTTSHVKPQHQMLFYKDDVLFYNISSMKSTESYFIPEVRIYDSGTYKCTVIVNNKEKTTAEYQVLVEGVPSPRVTLDKKEAIQGGIVRVNCSVPEEKAPIHFTIEKLELNEKMVKLKREKNSRDQNFVILEFPVEEQDRVLSFRCQARIISGIHMQTSESTKSELVTVTESFSTPKFHISPTGMIMEGAQLHIKCTIQVTHLAQEFPEIIIQKDKAIVAHNRHGNKAVYSVMAMVEHSGNYTCKVESSRISKVSSIVVNITELFSKPELESSFTHLDQGERLNLSCSIPGAPPANFTIQKEDTIVSQTQDFTKIASKSDSGTYICTAGIDKVVKKSNTVQIVVCEMLSQPRISYDAQFEVIKGQTIEVRCESISGTLPISYQLLKTSKVLENSTKNSNDPAVFKDNPTEDVEYQCVADNCHSHAKMLSEVLRVKVIAPVDEVQISILSSKVVESGEDIVLQCAVNEGSGPITYKFYREKEGKPFYQMTSNATQAFWTKQKASKEQEGEYYCTAFNRANHASSVPRSKILTVRVILAPWKKGLIAVVIIGVIIALLIIAAKCYFLRKAKAKQMPVEMSRPAVPLLNSNNEKMSDPNMEANSHYGHNDDVRNHAMKPINDNKEPLNSDVQYTEVQVSSAESHK[DLGKKDTETVYSEVRK]AVPDAVESRYSRTEGSLDGT
Red-highlighted residues are the mutants C622A, Y663F, Y690F, S700F, Y713F studied. In previously published work (Paddock et al 2011)1, Y690 is Y663 and Y713 is Y686. The difference is explained by the length of the signal peptide.
Signal peptide (27 amino acids).
The sequence in italics has been suggested to associate with the inner leaflet of the bilayer and to be releasable for regulated phosphorylation (Paddock et al 2011)1 .
Highly conserved and may be PKC phosphorylated (Osawa et al 1997)2. Underlined S residues are suggested to be phosphorylated (Paddock et al 2011)1.
[] indicates Exon 14 deletion (Osawa et al 1997)2 that may extend to AVP (see (b)).
Transmembrane
617-711 contains dual ITIM (immunoreceptor tyrosine-based inhibitory motif) and interacts with SHP2 (Src homology 2 domain-containing protein tyrosine phosphatase).
b
KCYFLRKAKAKQMPVEMSRPAVPLLNSNNEKMSDPNMEANSHYGHNDDVRNHAMKPINDNKEPLNSDVQYTEVQVSSAESHK[ENGRLP-COOH]DLGKKDTETVYSEVRKAVP[ENGRLP-COOH]DAVESRYSRTEGSLDGT-COOH
Exon 9 Exon 10 Exon 11 Exon 12 Exon 13 D14,15 D15 Exon 14 Exon 15 Exon 16
-COOH carboxy terminus
Supplementary Figure 11. PECAM1 amino acid sequence information in support of data in Figure 4
(a) Single-letter amino acid code for human PECAM1 (NP_000433.4), which comprises 738 amino acids and has a predicted mass of 82,522 Da. The colour-coding and formatting are explained in the accompanying text. References specified in the figure are Paddock et al 2011 1 and Osawa et al 1997 2.
(b) Amino acid sequence indicating potential C-terminal splice variants of PECAM1. The colour-coding is explained by the accompanying text. The abundance of splices in endothelial cells has been described by Chiu et al 2011 3. Exon 9 also encodes the transmembrane domain and 8 amino acids of the extracellular N-terminus. Interaction of Exon 13 with γ-catenin has been suggested (PMID: 9084985). Interaction of Exon 15 with β-catenin has been suggested, affecting b-catenin phosphorylation (Park et al 2015) 4.

## Slide 14
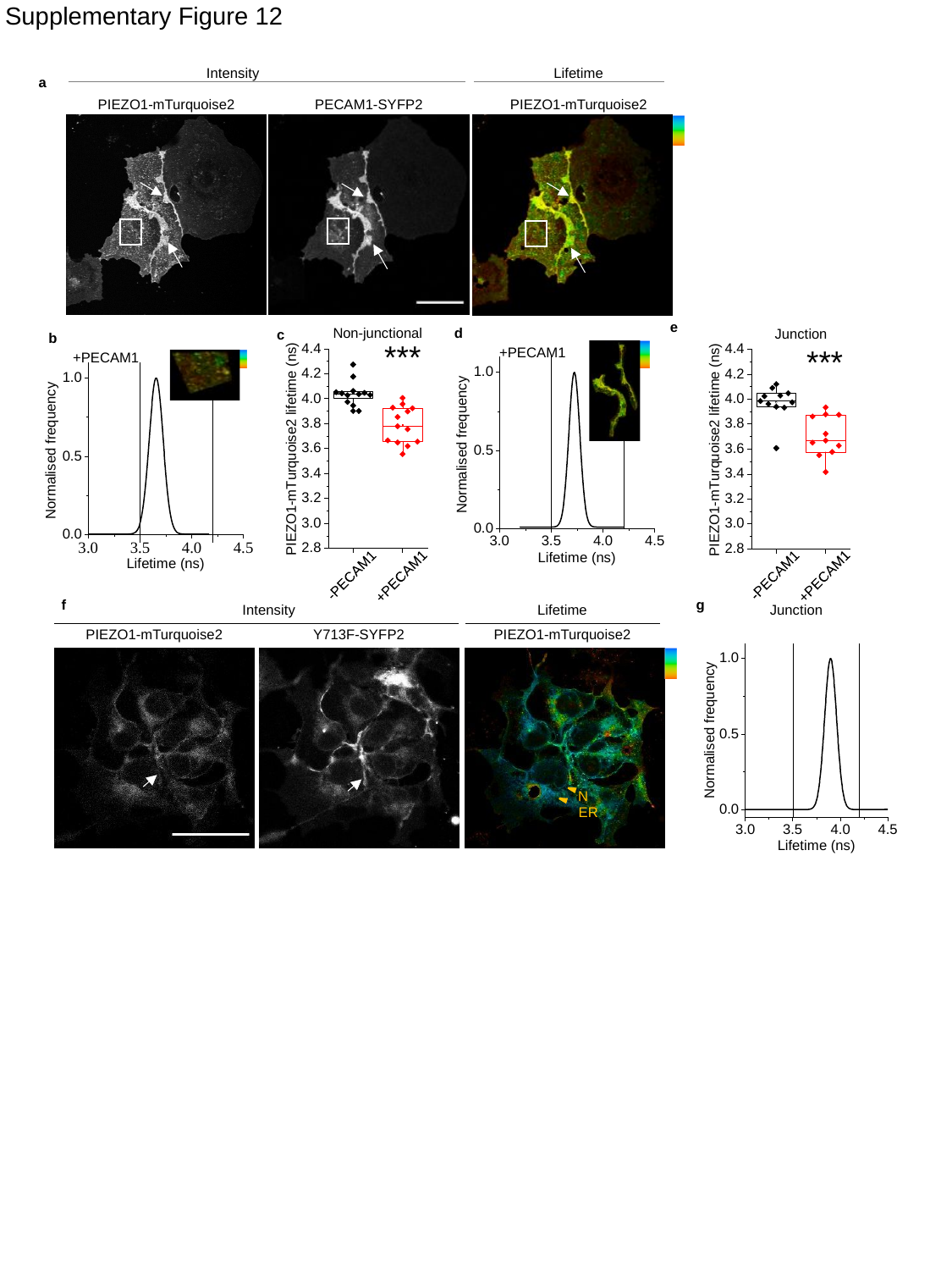

Supplementary Figure 12
Intensity
Lifetime
a
PIEZO1-mTurquoise2
PECAM1-SYFP2
PIEZO1-mTurquoise2
e
d
c
b
f
g
Intensity
Lifetime
Junction
PIEZO1-mTurquoise2
Y713F-SYFP2
PIEZO1-mTurquoise2
N
ER

## Slide 15
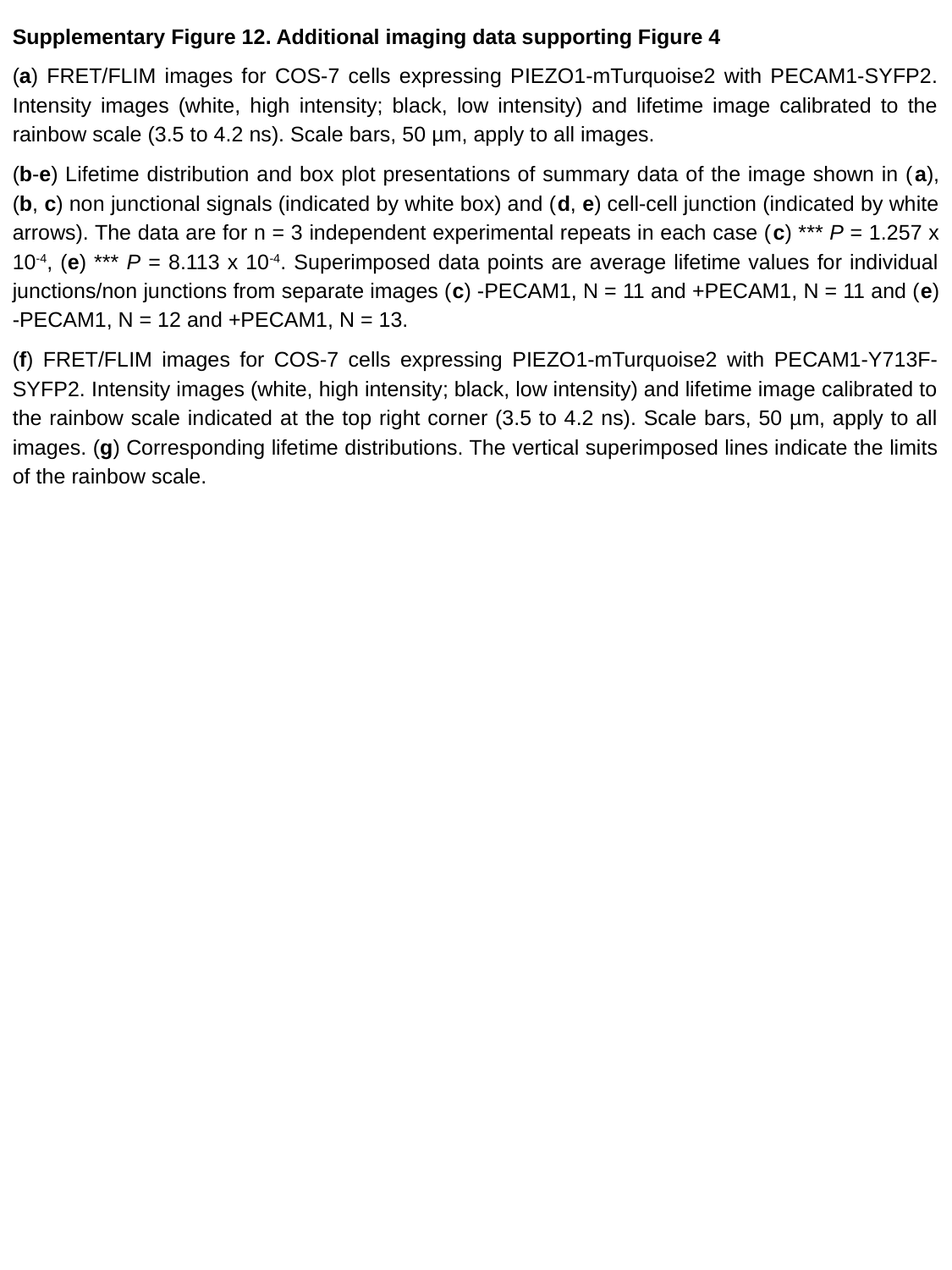

Supplementary Figure 12. Additional imaging data supporting Figure 4
(a) FRET/FLIM images for COS-7 cells expressing PIEZO1-mTurquoise2 with PECAM1-SYFP2. Intensity images (white, high intensity; black, low intensity) and lifetime image calibrated to the rainbow scale (3.5 to 4.2 ns). Scale bars, 50 µm, apply to all images.
(b-e) Lifetime distribution and box plot presentations of summary data of the image shown in (a), (b, c) non junctional signals (indicated by white box) and (d, e) cell-cell junction (indicated by white arrows). The data are for n = 3 independent experimental repeats in each case (c) *** P = 1.257 x 10^-4^, (e) *** P = 8.113 x 10^-4^. Superimposed data points are average lifetime values for individual junctions/non junctions from separate images (c) -PECAM1, N = 11 and +PECAM1, N = 11 and (e) -PECAM1, N = 12 and +PECAM1, N = 13.
(f) FRET/FLIM images for COS-7 cells expressing PIEZO1-mTurquoise2 with PECAM1-Y713F-SYFP2. Intensity images (white, high intensity; black, low intensity) and lifetime image calibrated to the rainbow scale indicated at the top right corner (3.5 to 4.2 ns). Scale bars, 50 µm, apply to all images. (g) Corresponding lifetime distributions. The vertical superimposed lines indicate the limits of the rainbow scale.

## Slide 16
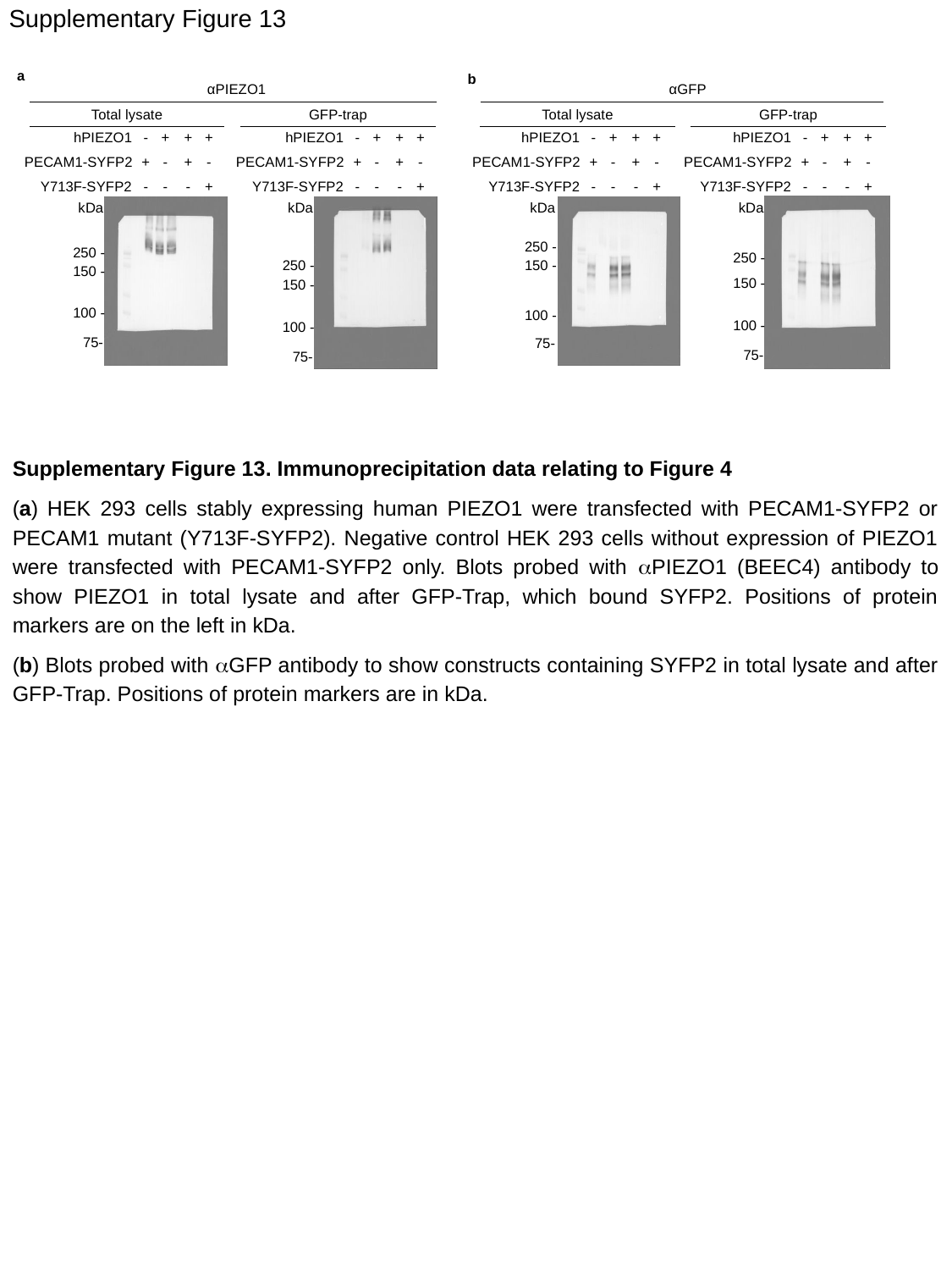

Supplementary Figure 13
a
b
αPIEZO1
αGFP
Total lysate
GFP-trap
Total lysate
GFP-trap
hPIEZO1
-
+
+
+
PECAM1-SYFP2
-
+
-
+
Y713F-SYFP2
-
-
-
+
kDa
250 -
150 -
100 -
75-
hPIEZO1
-
+
+
+
PECAM1-SYFP2
-
+
-
+
Y713F-SYFP2
-
-
-
+
kDa
250 -
150 -
100 -
75-
hPIEZO1
-
+
+
+
PECAM1-SYFP2
-
+
-
+
Y713F-SYFP2
-
-
-
+
kDa
250 -
150 -
100 -
75-
hPIEZO1
-
+
+
+
PECAM1-SYFP2
-
+
-
+
Y713F-SYFP2
-
-
-
+
kDa
250 -
150 -
100 -
75-
Supplementary Figure 13. Immunoprecipitation data relating to Figure 4
(a) HEK 293 cells stably expressing human PIEZO1 were transfected with PECAM1-SYFP2 or PECAM1 mutant (Y713F-SYFP2). Negative control HEK 293 cells without expression of PIEZO1 were transfected with PECAM1-SYFP2 only. Blots probed with αPIEZO1 (BEEC4) antibody to show PIEZO1 in total lysate and after GFP-Trap, which bound SYFP2. Positions of protein markers are on the left in kDa.
(b) Blots probed with αGFP antibody to show constructs containing SYFP2 in total lysate and after GFP-Trap. Positions of protein markers are in kDa.

## Slide 17
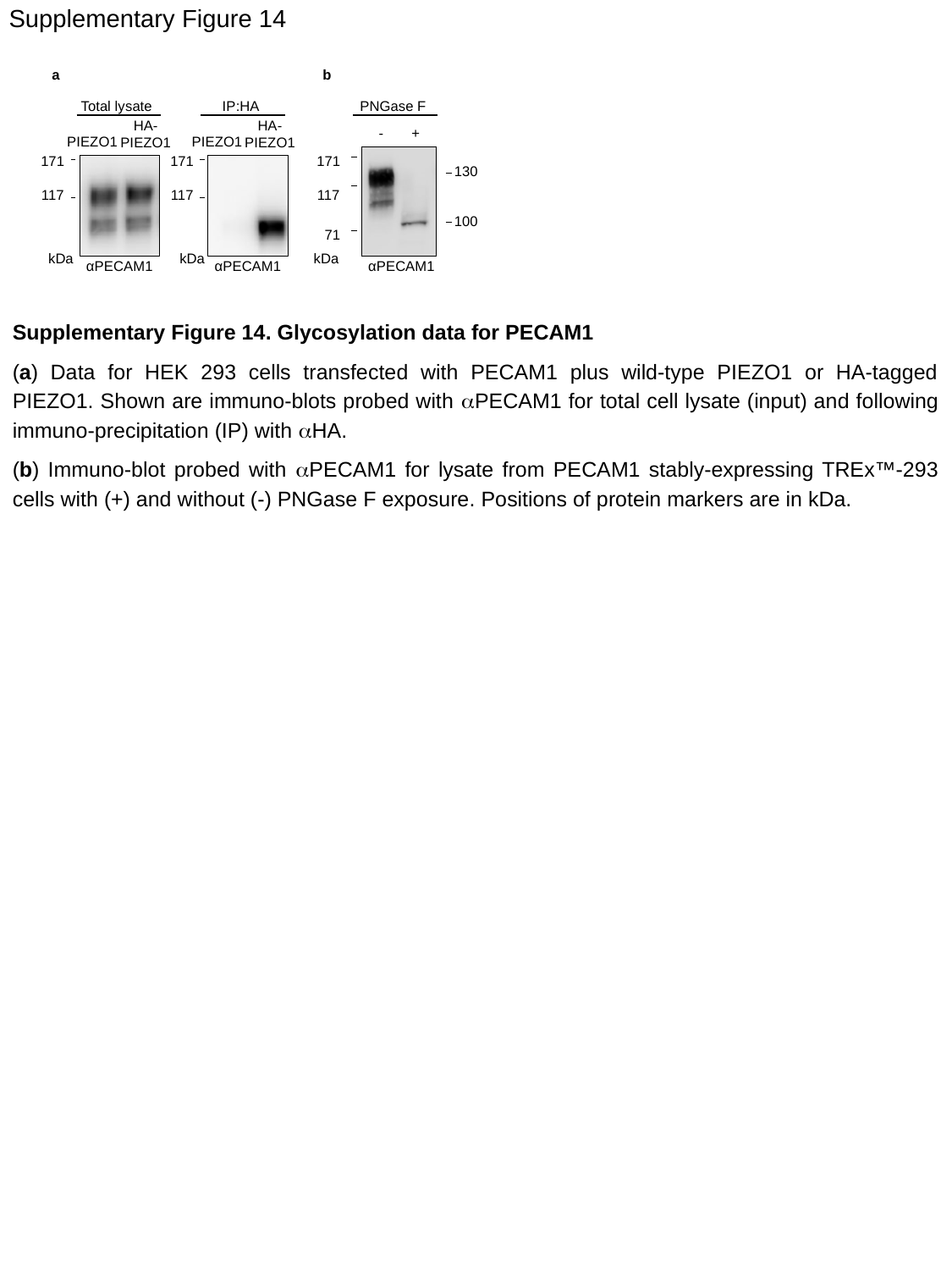

Supplementary Figure 14
a
b
Total lysate
IP:HA
HA-
PIEZO1
HA-
PIEZO1
PIEZO1
PIEZO1
171
171
117
117
kDa
kDa
αPECAM1
αPECAM1
PNGase F
- +
171
130
117
100
71
kDa
αPECAM1
Supplementary Figure 14. Glycosylation data for PECAM1
(a) Data for HEK 293 cells transfected with PECAM1 plus wild-type PIEZO1 or HA-tagged PIEZO1. Shown are immuno-blots probed with αPECAM1 for total cell lysate (input) and following immuno-precipitation (IP) with αHA.
(b) Immuno-blot probed with αPECAM1 for lysate from PECAM1 stably-expressing TREx™-293 cells with (+) and without (-) PNGase F exposure. Positions of protein markers are in kDa.

## Slide 18
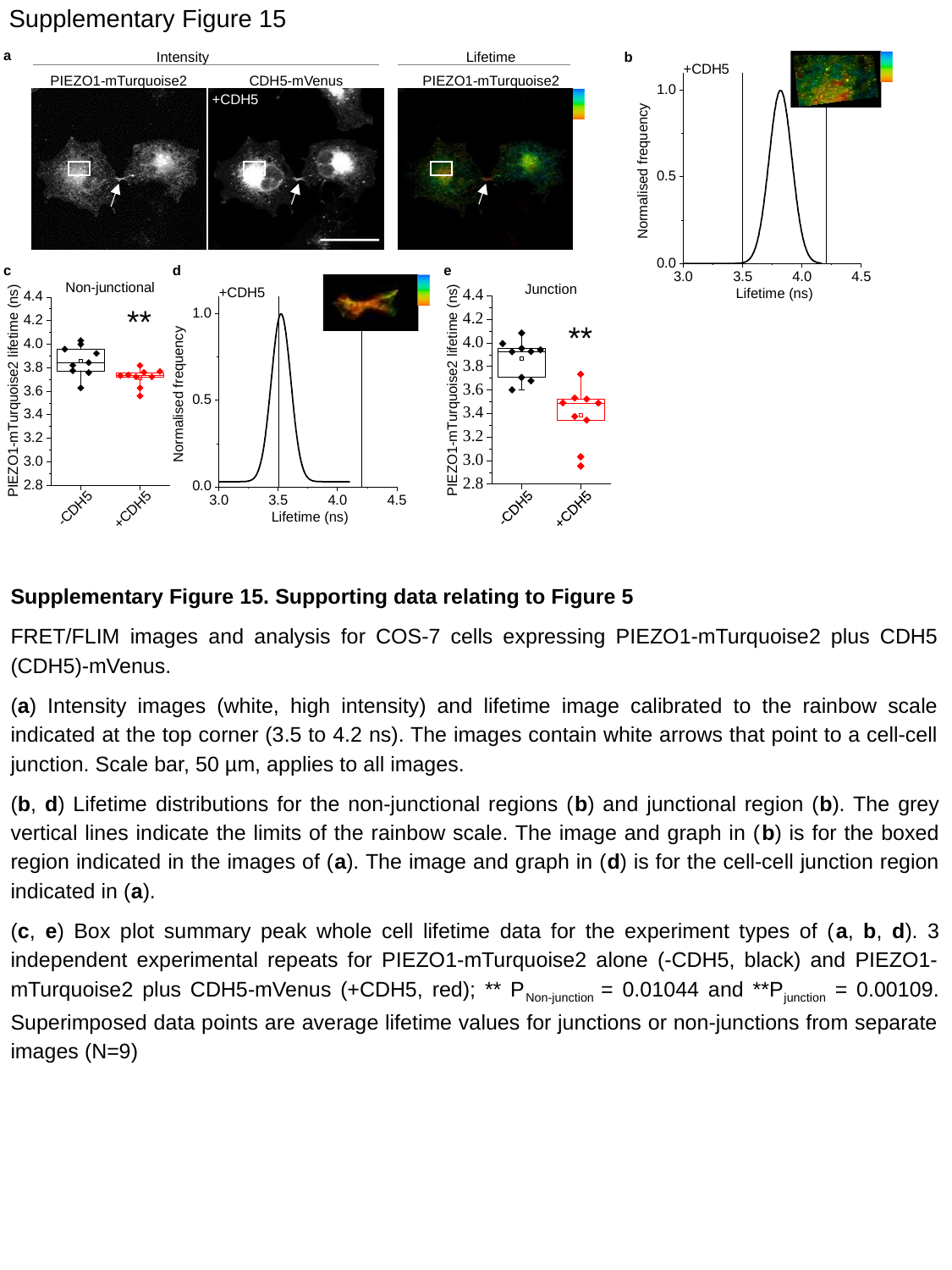

Supplementary Figure 15
a
b
Intensity
Lifetime
PIEZO1-mTurquoise2
CDH5-mVenus
PIEZO1-mTurquoise2
+CDH5
c
d
e
Supplementary Figure 15. Supporting data relating to Figure 5
FRET/FLIM images and analysis for COS-7 cells expressing PIEZO1-mTurquoise2 plus CDH5 (CDH5)-mVenus.
(a) Intensity images (white, high intensity) and lifetime image calibrated to the rainbow scale indicated at the top corner (3.5 to 4.2 ns). The images contain white arrows that point to a cell-cell junction. Scale bar, 50 µm, applies to all images.
(b, d) Lifetime distributions for the non-junctional regions (b) and junctional region (b). The grey vertical lines indicate the limits of the rainbow scale. The image and graph in (b) is for the boxed region indicated in the images of (a). The image and graph in (d) is for the cell-cell junction region indicated in (a).
(c, e) Box plot summary peak whole cell lifetime data for the experiment types of (a, b, d). 3 independent experimental repeats for PIEZO1-mTurquoise2 alone (-CDH5, black) and PIEZO1-mTurquoise2 plus CDH5-mVenus (+CDH5, red); ** PNon-junction = 0.01044 and **Pjunction = 0.00109. Superimposed data points are average lifetime values for junctions or non-junctions from separate images (N=9)

## Slide 19
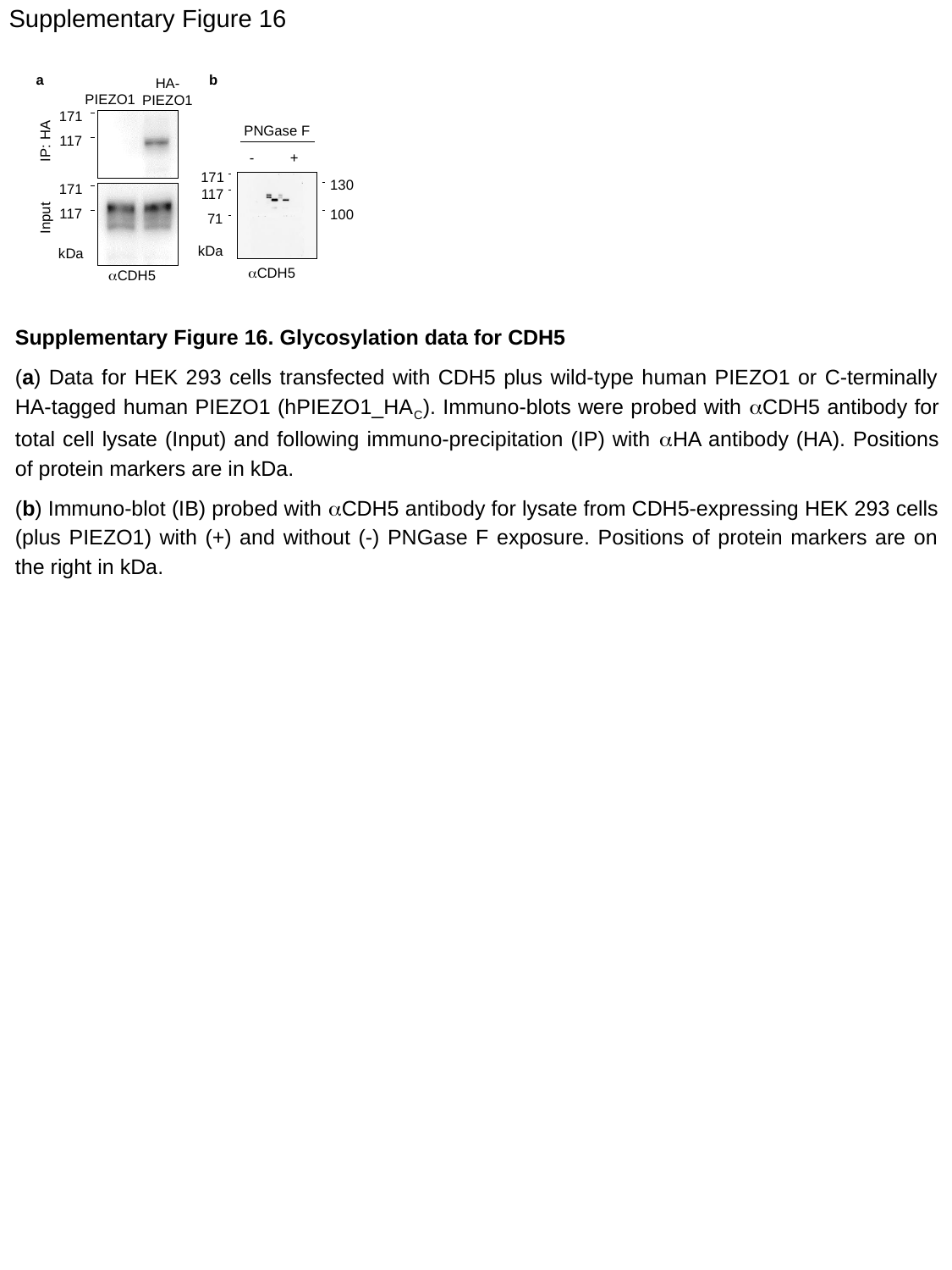

Supplementary Figure 16
a
b
HA-
PIEZO1
PIEZO1
171
PNGase F
117
IP: HA
- +
171
130
100
171
117
117
Input
71
kDa
kDa
αCDH5
αCDH5
Supplementary Figure 16. Glycosylation data for CDH5
(a) Data for HEK 293 cells transfected with CDH5 plus wild-type human PIEZO1 or C-terminally HA-tagged human PIEZO1 (hPIEZO1_HAC). Immuno-blots were probed with αCDH5 antibody for total cell lysate (Input) and following immuno-precipitation (IP) with αHA antibody (HA). Positions of protein markers are in kDa.
(b) Immuno-blot (IB) probed with αCDH5 antibody for lysate from CDH5-expressing HEK 293 cells (plus PIEZO1) with (+) and without (-) PNGase F exposure. Positions of protein markers are on the right in kDa.

## Slide 20
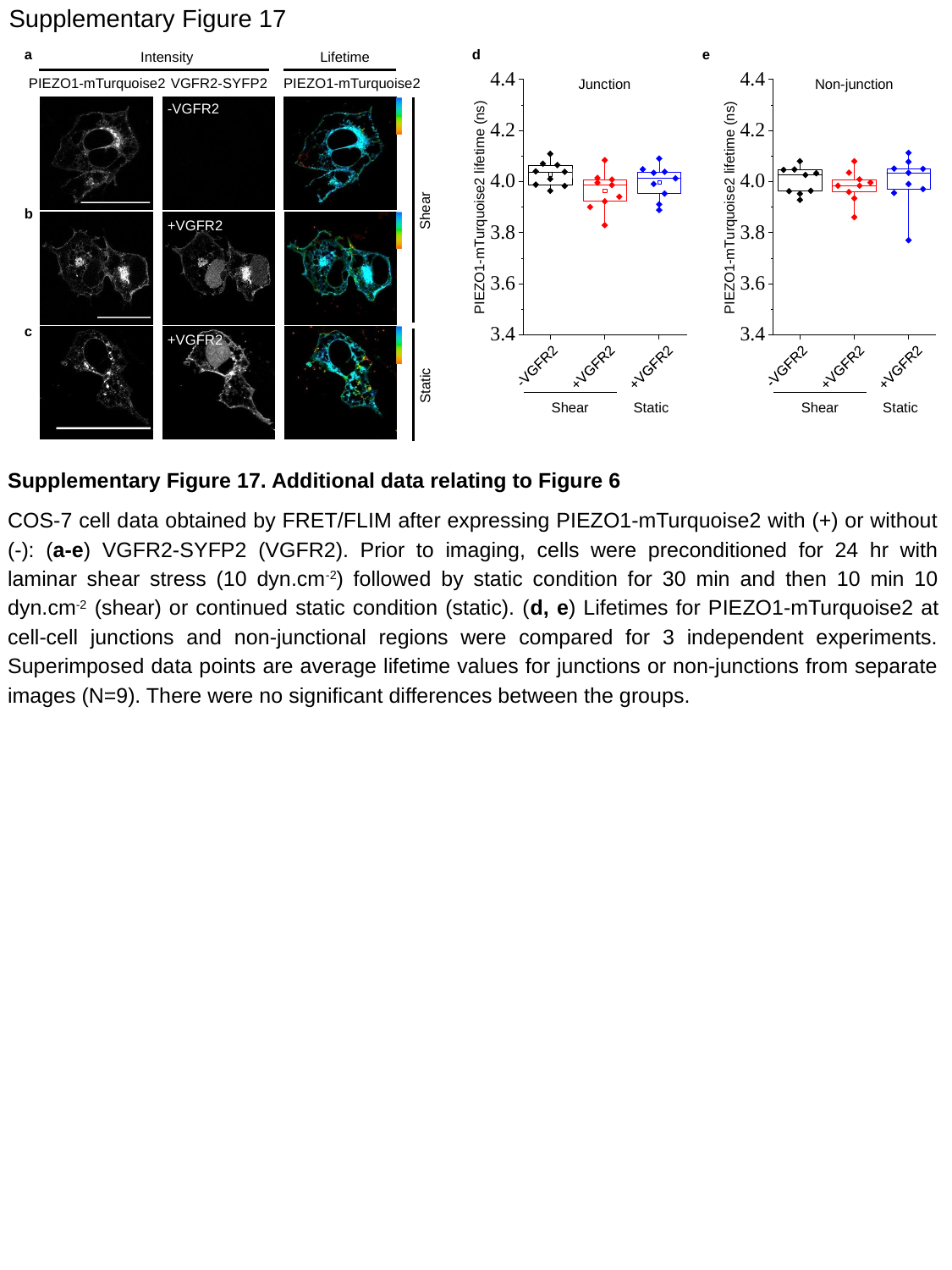

Supplementary Figure 17
a
d
e
Intensity
Lifetime
PIEZO1-mTurquoise2
VGFR2-SYFP2
PIEZO1-mTurquoise2
-VGFR2
Shear
b
+VGFR2
c
+VGFR2
Static
Supplementary Figure 17. Additional data relating to Figure 6
COS-7 cell data obtained by FRET/FLIM after expressing PIEZO1-mTurquoise2 with (+) or without (-): (a-e) VGFR2-SYFP2 (VGFR2). Prior to imaging, cells were preconditioned for 24 hr with laminar shear stress (10 dyn.cm-2) followed by static condition for 30 min and then 10 min 10 dyn.cm-2 (shear) or continued static condition (static). (d, e) Lifetimes for PIEZO1-mTurquoise2 at cell-cell junctions and non-junctional regions were compared for 3 independent experiments. Superimposed data points are average lifetime values for junctions or non-junctions from separate images (N=9). There were no significant differences between the groups.

## Slide 21
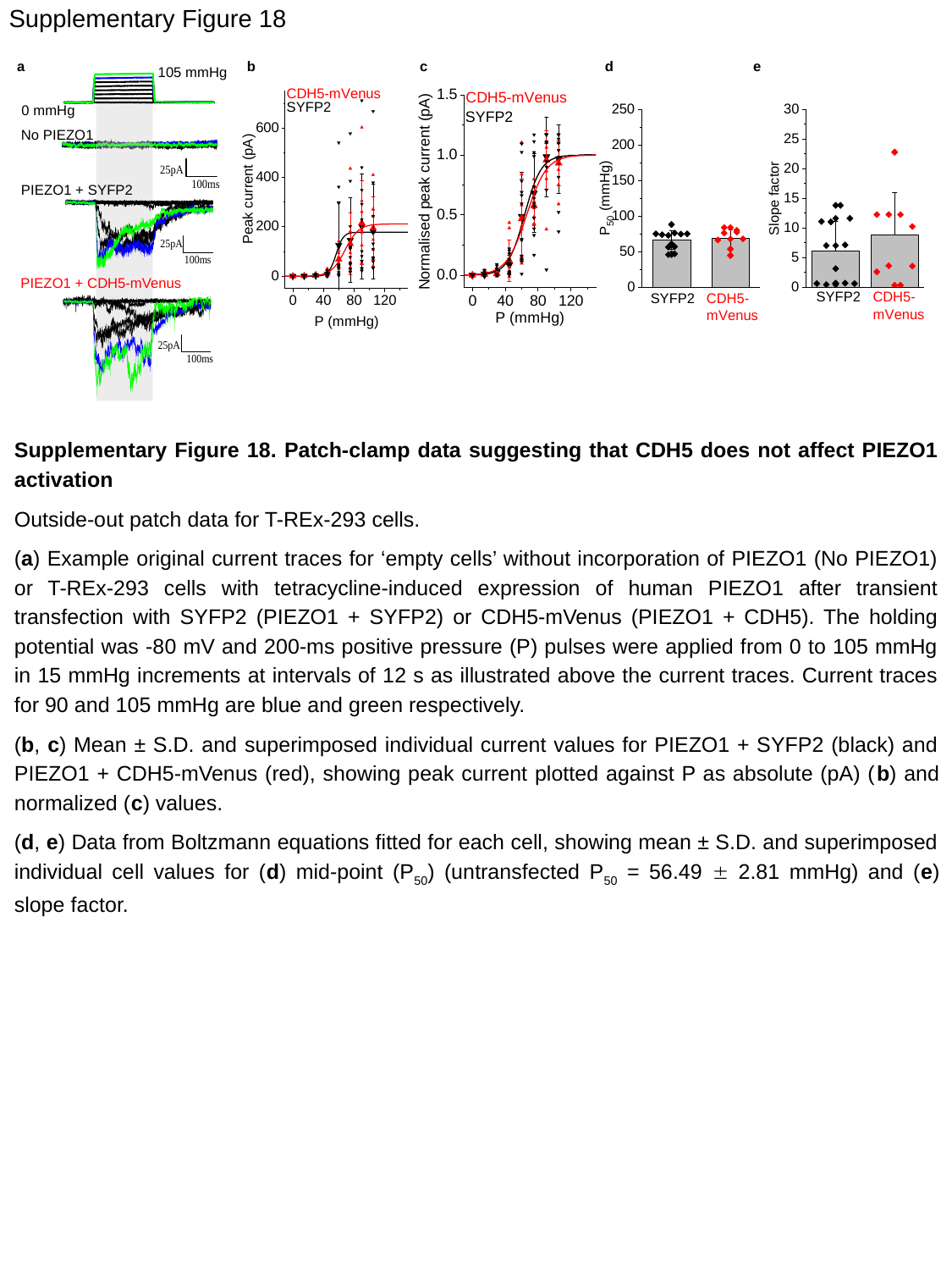

Supplementary Figure 18
a
b
c
d
e
105 mmHg
0 mmHg
No PIEZO1
PIEZO1 + SYFP2
PIEZO1 + CDH5-mVenus
Supplementary Figure 18. Patch-clamp data suggesting that CDH5 does not affect PIEZO1 activation
Outside-out patch data for T-REx-293 cells.
(a) Example original current traces for ‘empty cells’ without incorporation of PIEZO1 (No PIEZO1) or T-REx-293 cells with tetracycline-induced expression of human PIEZO1 after transient transfection with SYFP2 (PIEZO1 + SYFP2) or CDH5-mVenus (PIEZO1 + CDH5). The holding potential was -80 mV and 200-ms positive pressure (P) pulses were applied from 0 to 105 mmHg in 15 mmHg increments at intervals of 12 s as illustrated above the current traces. Current traces for 90 and 105 mmHg are blue and green respectively.
(b, c) Mean ± S.D. and superimposed individual current values for PIEZO1 + SYFP2 (black) and PIEZO1 + CDH5-mVenus (red), showing peak current plotted against P as absolute (pA) (b) and normalized (c) values.
(d, e) Data from Boltzmann equations fitted for each cell, showing mean ± S.D. and superimposed individual cell values for (d) mid-point (P50) (untransfected P50 = 56.49 ± 2.81 mmHg) and (e) slope factor.

## Slide 22
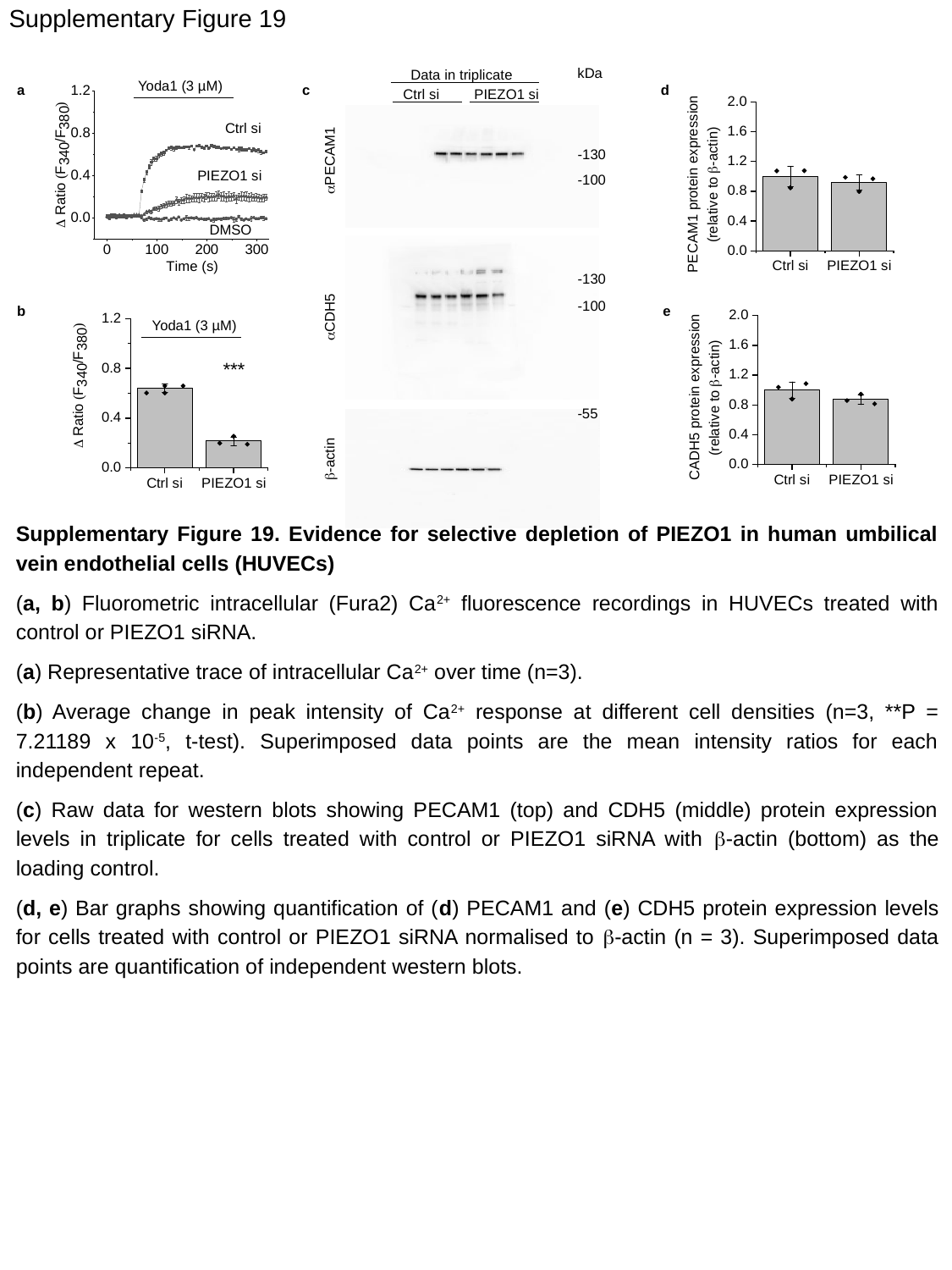

Supplementary Figure 19
kDa
Data in triplicate
Yoda1 (3 µM)
a
d
c
Ctrl si
PIEZO1 si
-130
αPECAM1
-100
-130
-100
b
e
αCDH5
Yoda1 (3 µM)
-55
β-actin
Supplementary Figure 19. Evidence for selective depletion of PIEZO1 in human umbilical vein endothelial cells (HUVECs)
(a, b) Fluorometric intracellular (Fura2) Ca2+ fluorescence recordings in HUVECs treated with control or PIEZO1 siRNA.
(a) Representative trace of intracellular Ca2+ over time (n=3).
(b) Average change in peak intensity of Ca2+ response at different cell densities (n=3, **P = 7.21189 x 10-5, t-test). Superimposed data points are the mean intensity ratios for each independent repeat.
(c) Raw data for western blots showing PECAM1 (top) and CDH5 (middle) protein expression levels in triplicate for cells treated with control or PIEZO1 siRNA with β-actin (bottom) as the loading control.
(d, e) Bar graphs showing quantification of (d) PECAM1 and (e) CDH5 protein expression levels for cells treated with control or PIEZO1 siRNA normalised to β-actin (n = 3). Superimposed data points are quantification of independent western blots.

## Slide 23
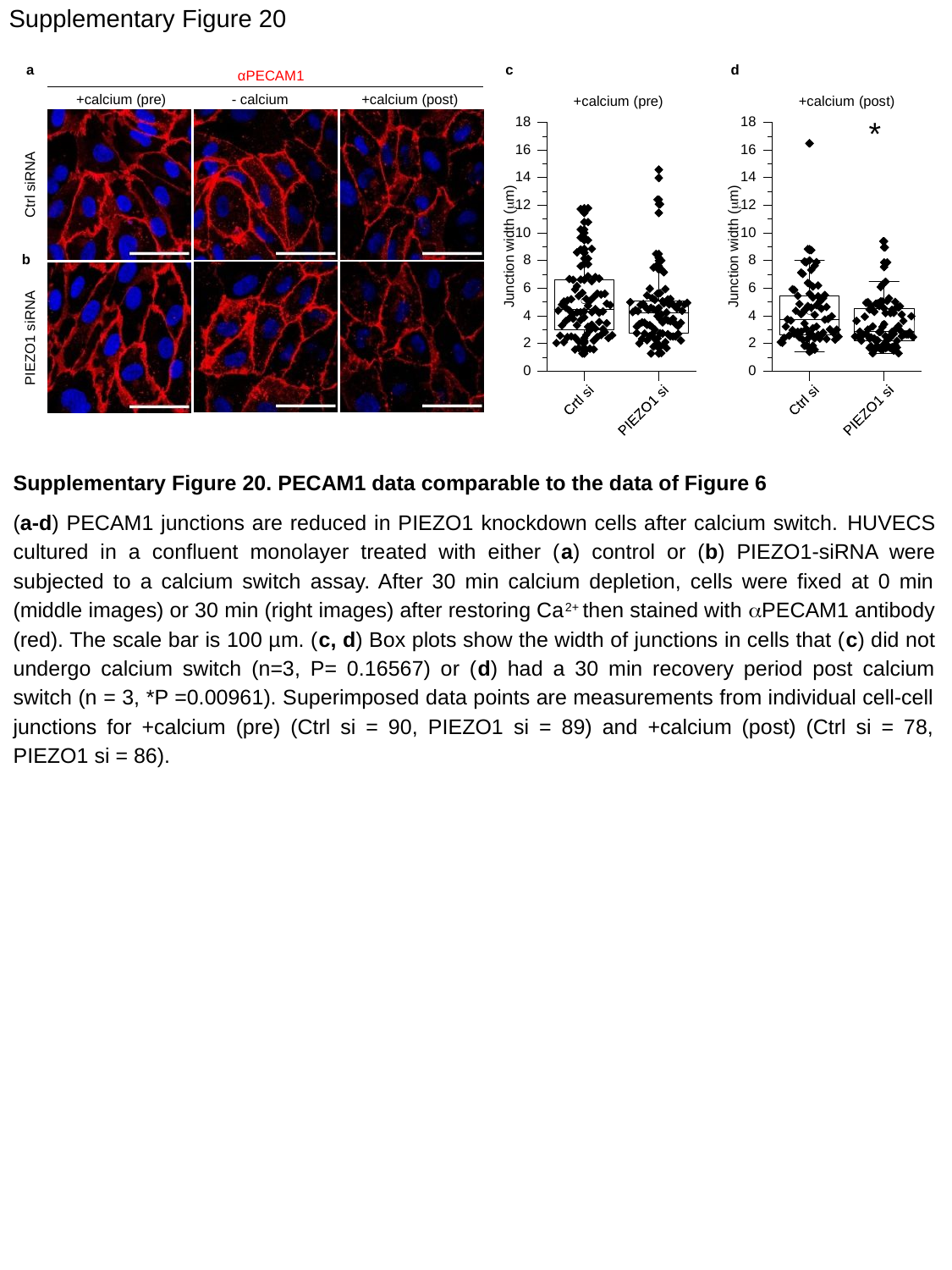

Supplementary Figure 20
a
c
d
αPECAM1
+calcium (pre)
 - calcium
+calcium (post)
Ctrl siRNA
b
PIEZO1 siRNA
Supplementary Figure 20. PECAM1 data comparable to the data of Figure 6
(a-d) PECAM1 junctions are reduced in PIEZO1 knockdown cells after calcium switch. HUVECS cultured in a confluent monolayer treated with either (a) control or (b) PIEZO1-siRNA were subjected to a calcium switch assay. After 30 min calcium depletion, cells were fixed at 0 min (middle images) or 30 min (right images) after restoring Ca2+ then stained with αPECAM1 antibody (red). The scale bar is 100 µm. (c, d) Box plots show the width of junctions in cells that (c) did not undergo calcium switch (n=3, P= 0.16567) or (d) had a 30 min recovery period post calcium switch (n = 3, *P =0.00961). Superimposed data points are measurements from individual cell-cell junctions for +calcium (pre) (Ctrl si = 90, PIEZO1 si = 89) and +calcium (post) (Ctrl si = 78, PIEZO1 si = 86).

## Slide 24
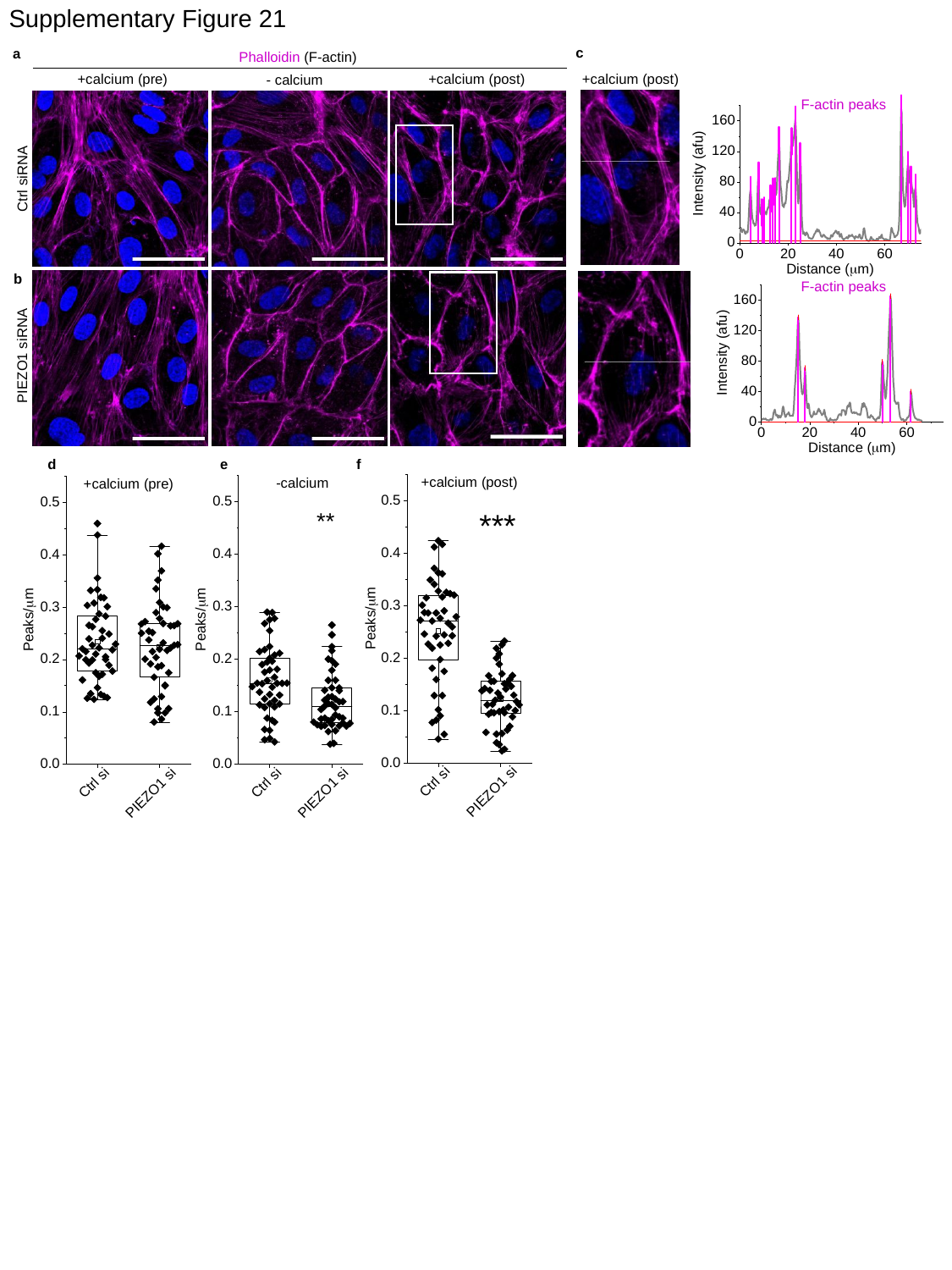

Supplementary Figure 21
c
a
Phalloidin (F-actin)
+calcium (pre)
+calcium (post)
+calcium (post)
 - calcium
F-actin peaks
Ctrl siRNA
Ctrl siRNA
b
F-actin peaks
PIEZO1 siRNA
PIEZO1 siRNA
d
e
f

## Slide 25
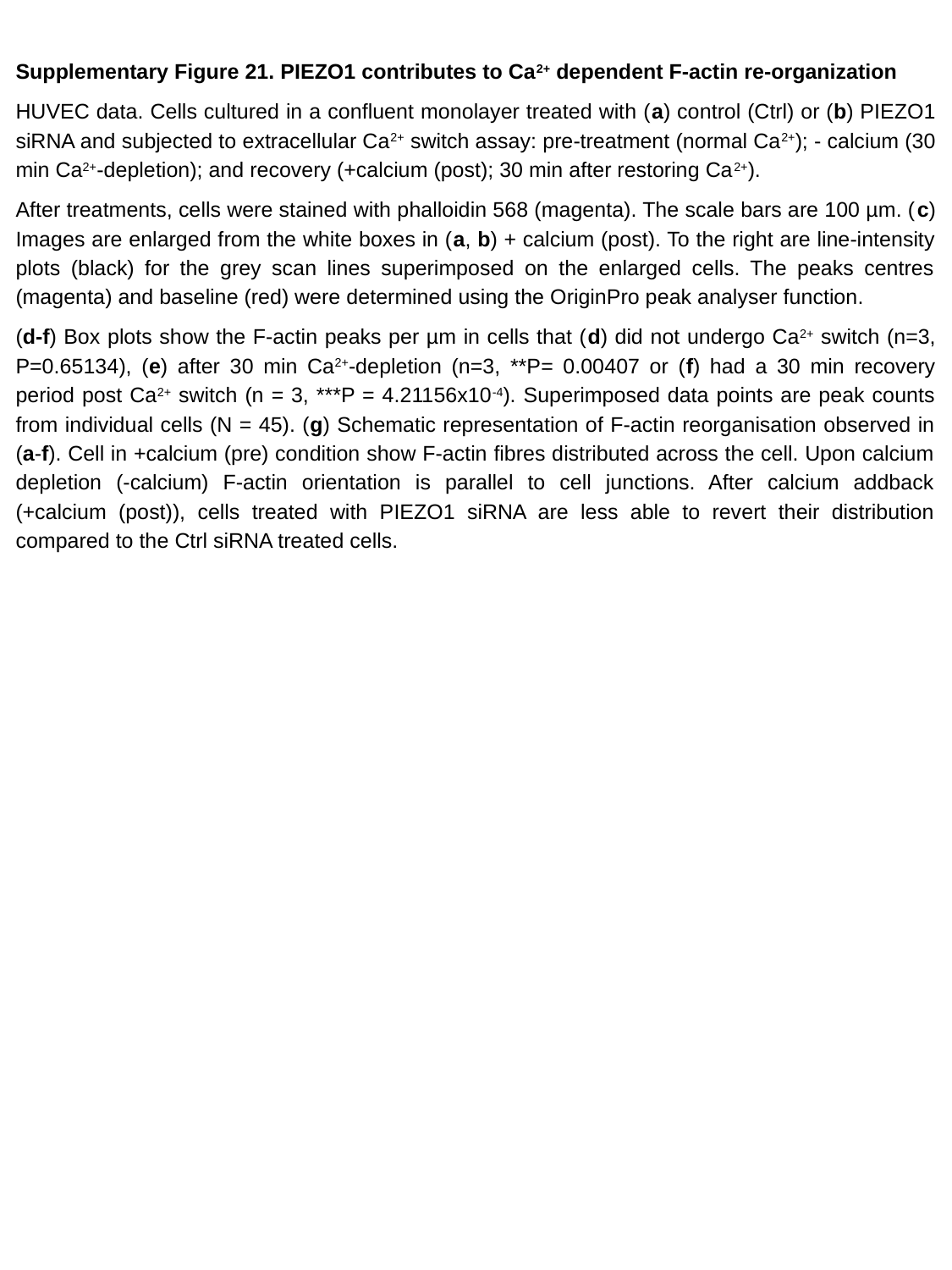

Supplementary Figure 21. PIEZO1 contributes to Ca2+ dependent F-actin re-organization
HUVEC data. Cells cultured in a confluent monolayer treated with (a) control (Ctrl) or (b) PIEZO1 siRNA and subjected to extracellular Ca2+ switch assay: pre-treatment (normal Ca2+); - calcium (30 min Ca2+-depletion); and recovery (+calcium (post); 30 min after restoring Ca2+).
After treatments, cells were stained with phalloidin 568 (magenta). The scale bars are 100 µm. (c) Images are enlarged from the white boxes in (a, b) + calcium (post). To the right are line-intensity plots (black) for the grey scan lines superimposed on the enlarged cells. The peaks centres (magenta) and baseline (red) were determined using the OriginPro peak analyser function.
(d-f) Box plots show the F-actin peaks per µm in cells that (d) did not undergo Ca2+ switch (n=3, P=0.65134), (e) after 30 min Ca2+-depletion (n=3, **P= 0.00407 or (f) had a 30 min recovery period post Ca2+ switch (n = 3, ***P = 4.21156x10-4). Superimposed data points are peak counts from individual cells (N = 45). (g) Schematic representation of F-actin reorganisation observed in (a-f). Cell in +calcium (pre) condition show F-actin fibres distributed across the cell. Upon calcium depletion (-calcium) F-actin orientation is parallel to cell junctions. After calcium addback (+calcium (post)), cells treated with PIEZO1 siRNA are less able to revert their distribution compared to the Ctrl siRNA treated cells.

## Slide 26
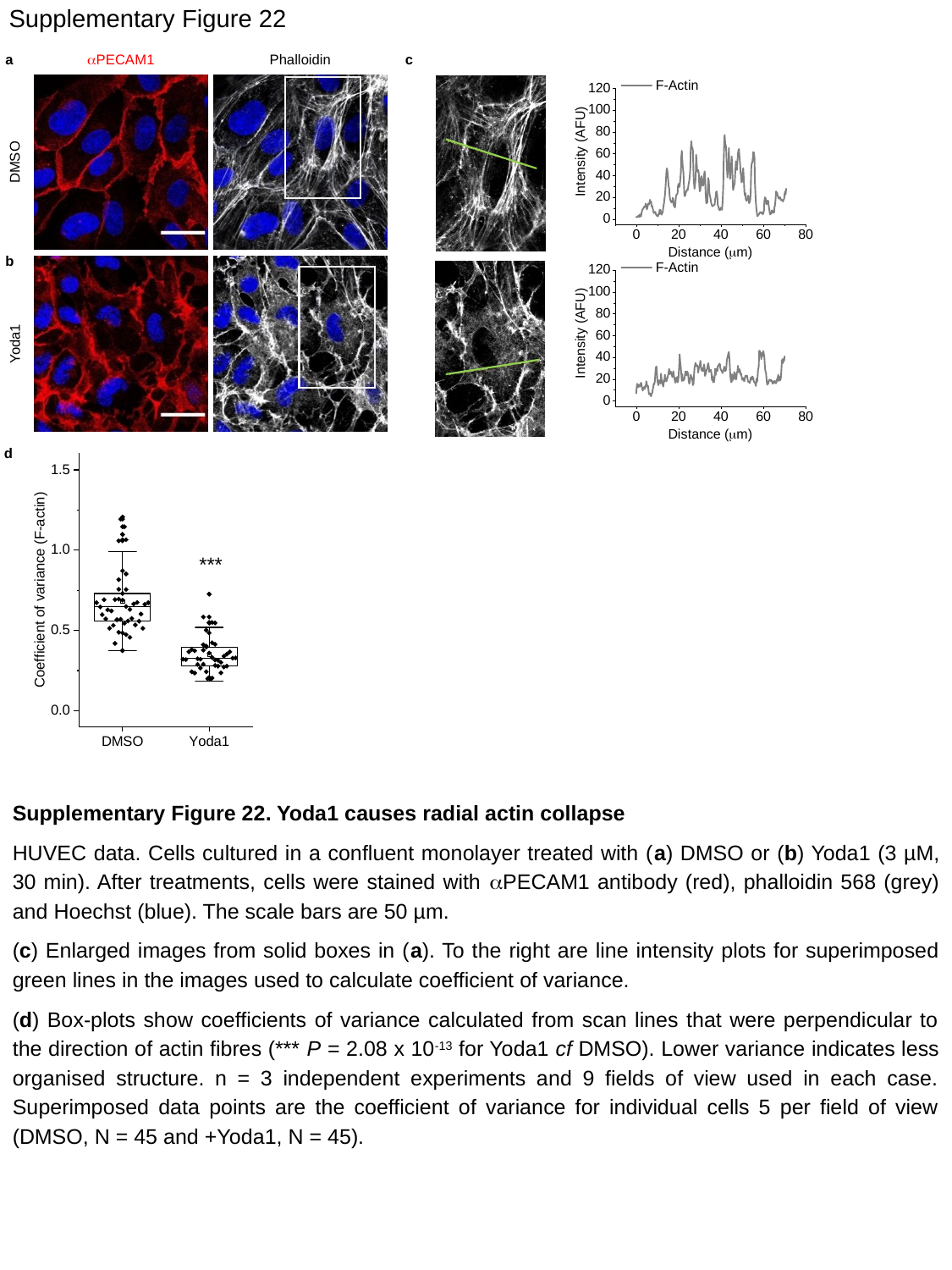

Supplementary Figure 22
a
αPECAM1
Phalloidin
c
DMSO
b
Yoda1
d
Supplementary Figure 22. Yoda1 causes radial actin collapse
HUVEC data. Cells cultured in a confluent monolayer treated with (a) DMSO or (b) Yoda1 (3 µM, 30 min). After treatments, cells were stained with αPECAM1 antibody (red), phalloidin 568 (grey) and Hoechst (blue). The scale bars are 50 µm.
(c) Enlarged images from solid boxes in (a). To the right are line intensity plots for superimposed green lines in the images used to calculate coefficient of variance.
(d) Box-plots show coefficients of variance calculated from scan lines that were perpendicular to the direction of actin fibres (*** P = 2.08 x 10^-13^ for Yoda1 cf DMSO). Lower variance indicates less organised structure. n = 3 independent experiments and 9 fields of view used in each case. Superimposed data points are the coefficient of variance for individual cells 5 per field of view (DMSO, N = 45 and +Yoda1, N = 45).

## Slide 27
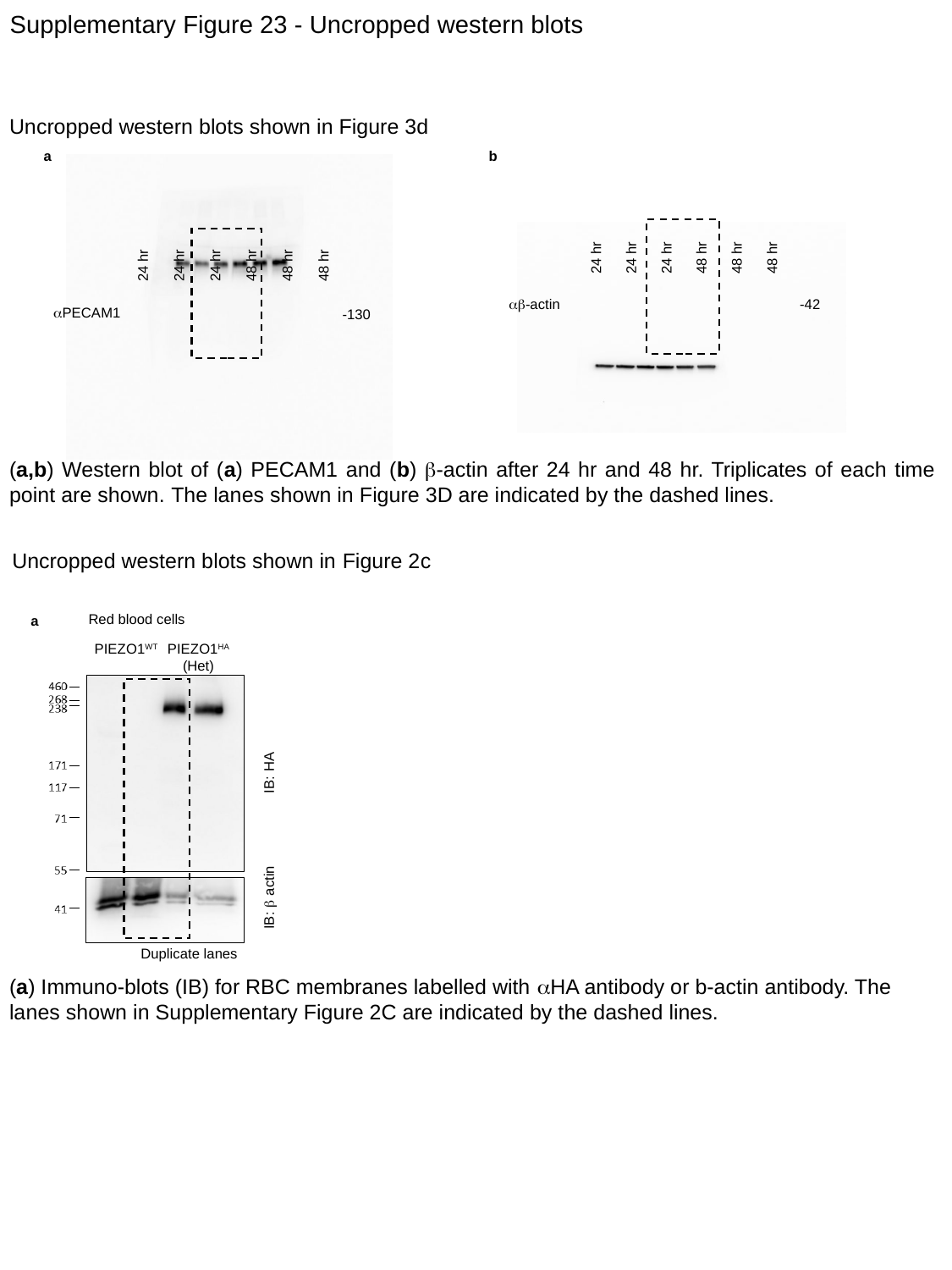

Supplementary Figure 23 - Uncropped western blots
Uncropped western blots shown in Figure 3d
a
b
αβ-actin
-42
24 hr
24 hr
24 hr
48 hr
48 hr
48 hr
24 hr
24 hr
24 hr
48 hr
48 hr
48 hr
αPECAM1
-130
(a,b) Western blot of (a) PECAM1 and (b) β-actin after 24 hr and 48 hr. Triplicates of each time point are shown. The lanes shown in Figure 3D are indicated by the dashed lines.
Uncropped western blots shown in Figure 2c
Red blood cells
a
PIEZO1WT
PIEZO1HA
(Het)
IB: HA
IB: β actin
Duplicate lanes
(a) Immuno-blots (IB) for RBC membranes labelled with αHA antibody or b-actin antibody. The lanes shown in Supplementary Figure 2C are indicated by the dashed lines.

## Slide 28
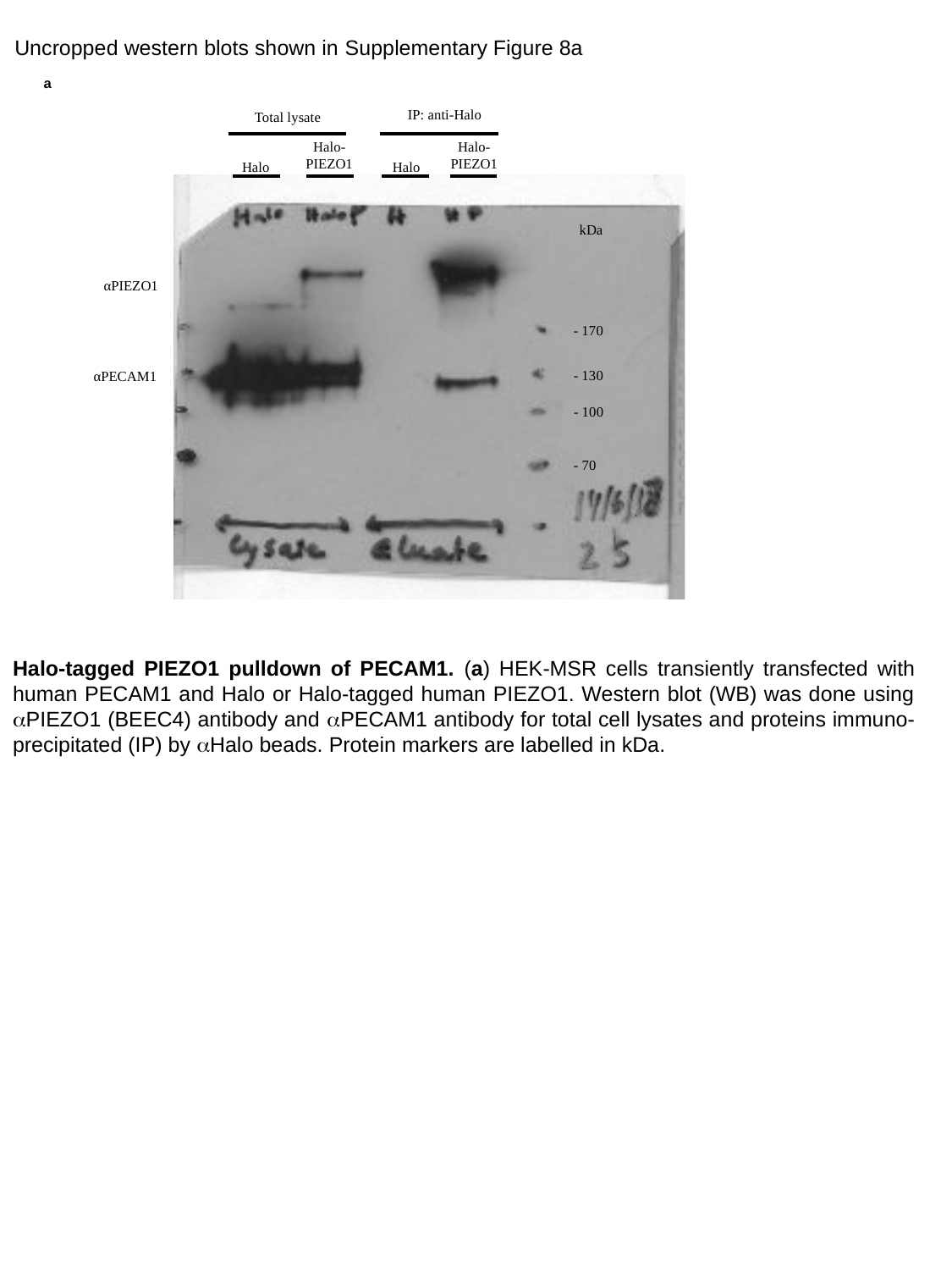

Uncropped western blots shown in Supplementary Figure 8a
a
IP: anti-Halo
Total lysate
Halo-
PIEZO1
Halo-
PIEZO1
Halo
Halo
kDa
αPIEZO1
- 170
- 130
αPECAM1
- 100
- 70
Halo-tagged PIEZO1 pulldown of PECAM1. (a) HEK-MSR cells transiently transfected with human PECAM1 and Halo or Halo-tagged human PIEZO1. Western blot (WB) was done using αPIEZO1 (BEEC4) antibody and αPECAM1 antibody for total cell lysates and proteins immuno-precipitated (IP) by αHalo beads. Protein markers are labelled in kDa.

## Slide 29
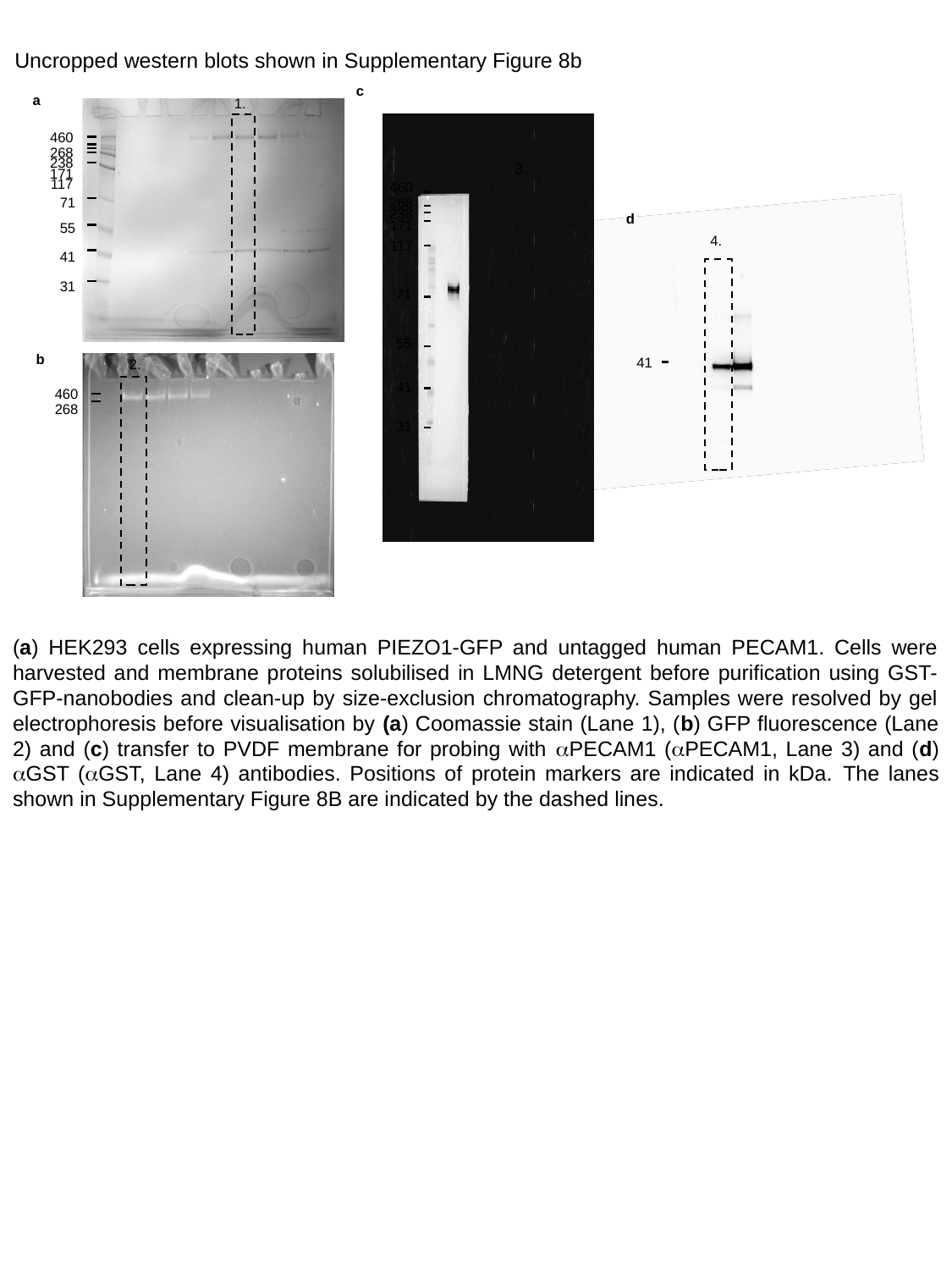

Uncropped western blots shown in Supplementary Figure 8b
c
a
1.
3.
460
268
238
171
117
71
55
41
31
460
268
238
171
117
71
d
55
4.
41
31
b
41
2.
460
268
(a) HEK293 cells expressing human PIEZO1-GFP and untagged human PECAM1. Cells were harvested and membrane proteins solubilised in LMNG detergent before purification using GST-GFP-nanobodies and clean-up by size-exclusion chromatography. Samples were resolved by gel electrophoresis before visualisation by (a) Coomassie stain (Lane 1), (b) GFP fluorescence (Lane 2) and (c) transfer to PVDF membrane for probing with αPECAM1 (aPECAM1, Lane 3) and (d) αGST (aGST, Lane 4) antibodies. Positions of protein markers are indicated in kDa. The lanes shown in Supplementary Figure 8B are indicated by the dashed lines.

## Slide 30
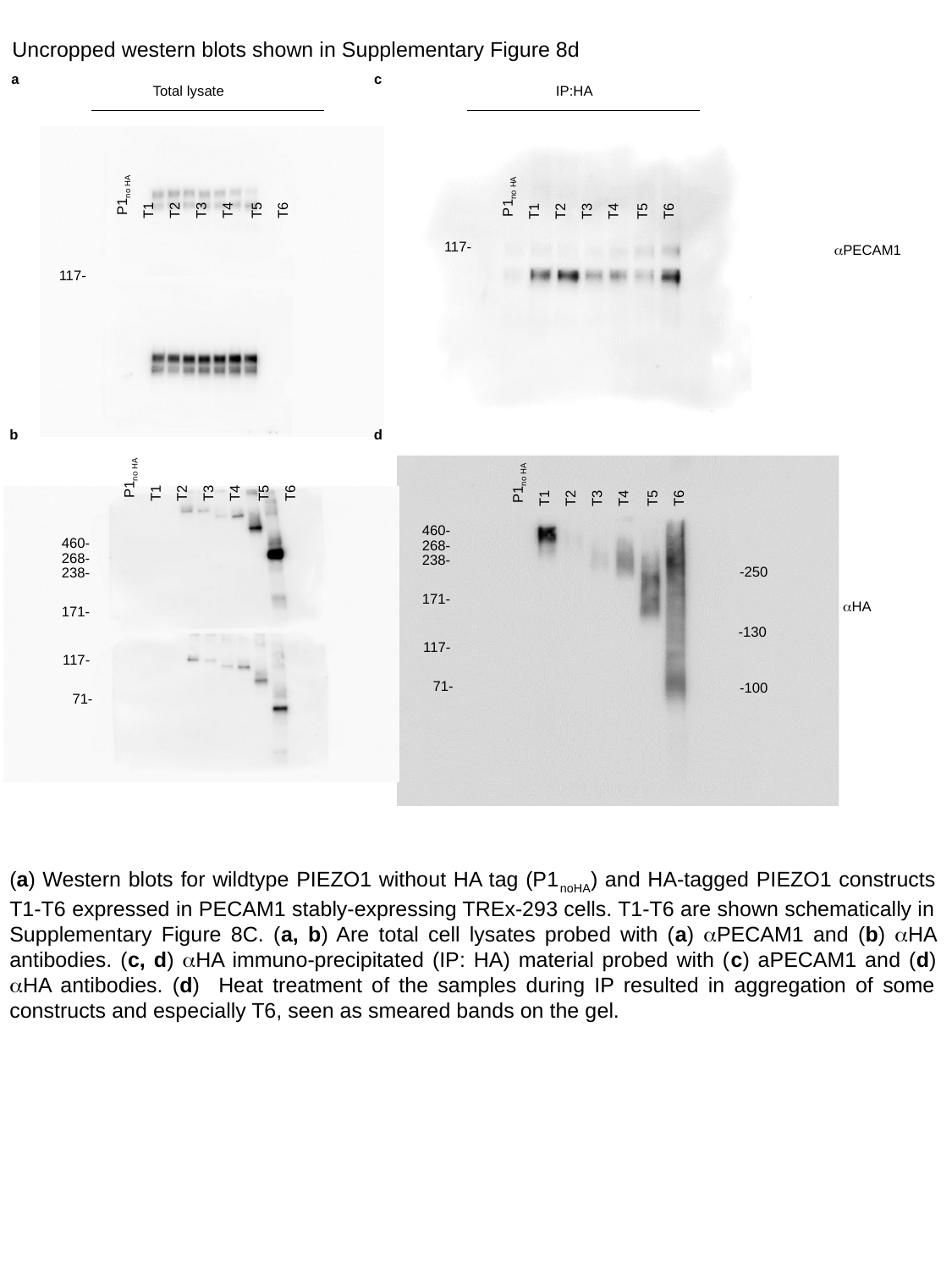

Uncropped western blots shown in Supplementary Figure 8d
a
c
Total lysate
IP:HA
P1no HA
T3
T1
T2
T4
T5
T6
P1no HA
T3
T1
T2
T4
T5
T6
117-
αPECAM1
117-
b
d
P1no HA
T3
T1
T2
T4
T5
T6
P1no HA
T3
T1
T2
T4
T5
T6
460-
268-
238-
171-
117-
71-
460-
268-
238-
171-
117-
71-
-250
αHA
-130
-100
(a) Western blots for wildtype PIEZO1 without HA tag (P1noHA) and HA-tagged PIEZO1 constructs T1-T6 expressed in PECAM1 stably-expressing TREx-293 cells. T1-T6 are shown schematically in Supplementary Figure 8C. (a, b) Are total cell lysates probed with (a) αPECAM1 and (b) αHA antibodies. (c, d) αHA immuno-precipitated (IP: HA) material probed with (c) aPECAM1 and (d) αHA antibodies. (d) Heat treatment of the samples during IP resulted in aggregation of some constructs and especially T6, seen as smeared bands on the gel.

## Slide 31
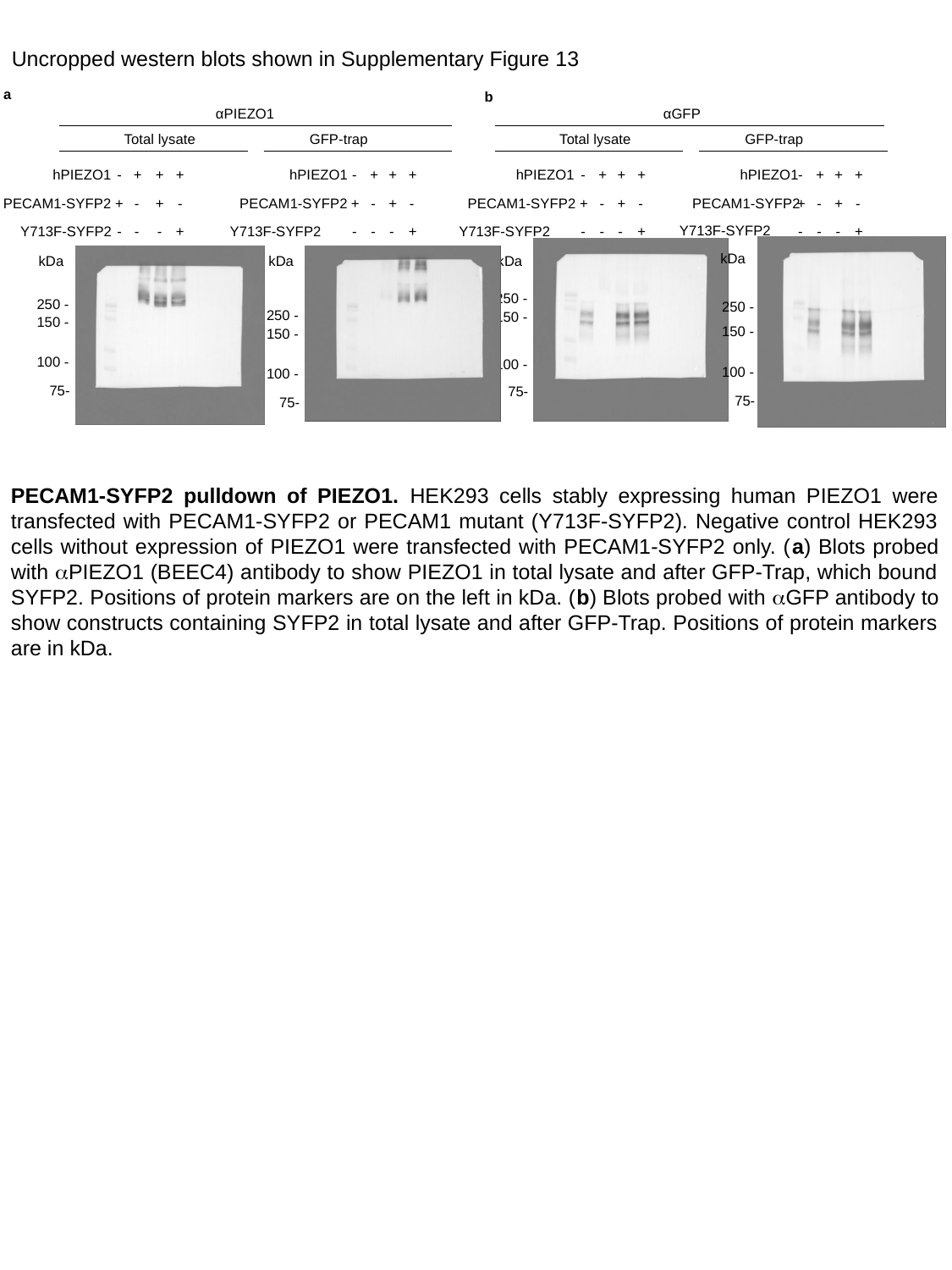

Uncropped western blots shown in Supplementary Figure 13
a
αPIEZO1
αGFP
Total lysate
GFP-trap
Total lysate
GFP-trap
hPIEZO1
-
+
+
+
hPIEZO1
-
+
+
+
+
-
+
-
PECAM1-SYFP2
-
-
-
+
Y713F-SYFP2
hPIEZO1
-
+
+
+
+
-
+
-
PECAM1-SYFP2
-
-
-
+
Y713F-SYFP2
hPIEZO1
-
+
+
+
+
-
+
-
PECAM1-SYFP2
Y713F-SYFP2
-
-
-
+
+
-
+
-
PECAM1-SYFP2
Y713F-SYFP2
-
-
-
+
kDa
250 -
150 -
100 -
75-
kDa
250 -
150 -
100 -
75-
kDa
250 -
150 -
100 -
75-
kDa
250 -
150 -
100 -
75-
b
PECAM1-SYFP2 pulldown of PIEZO1. HEK293 cells stably expressing human PIEZO1 were transfected with PECAM1-SYFP2 or PECAM1 mutant (Y713F-SYFP2). Negative control HEK293 cells without expression of PIEZO1 were transfected with PECAM1-SYFP2 only. (a) Blots probed with αPIEZO1 (BEEC4) antibody to show PIEZO1 in total lysate and after GFP-Trap, which bound SYFP2. Positions of protein markers are on the left in kDa. (b) Blots probed with αGFP antibody to show constructs containing SYFP2 in total lysate and after GFP-Trap. Positions of protein markers are in kDa.

## Slide 32
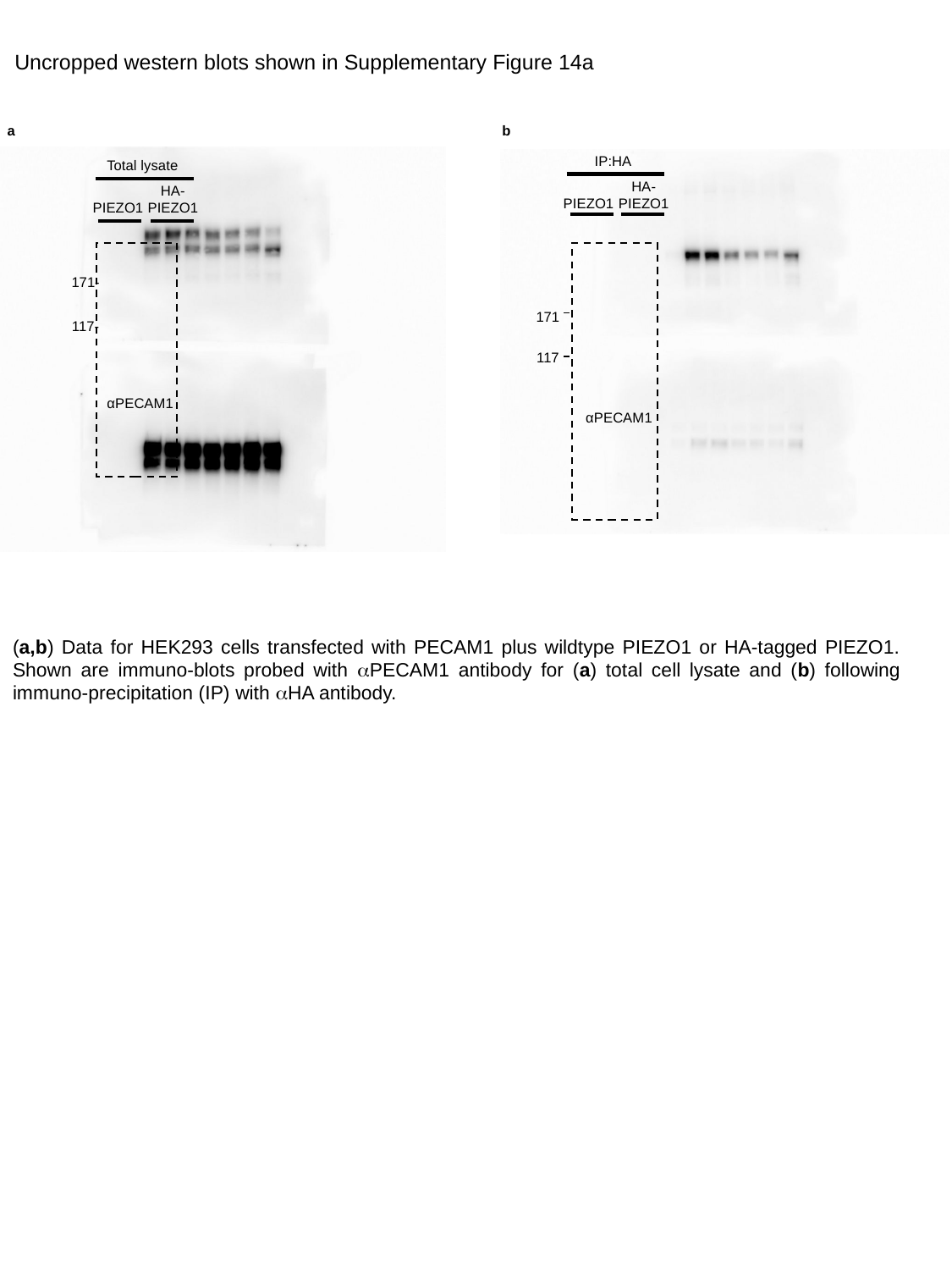

Uncropped western blots shown in Supplementary Figure 14a
a
b
IP:HA
HA-
PIEZO1
PIEZO1
171-
117-
Total lysate
HA-
PIEZO1
PIEZO1
171
117
αPECAM1
αPECAM1
(a,b) Data for HEK293 cells transfected with PECAM1 plus wildtype PIEZO1 or HA-tagged PIEZO1. Shown are immuno-blots probed with αPECAM1 antibody for (a) total cell lysate and (b) following immuno-precipitation (IP) with αHA antibody.

## Slide 33
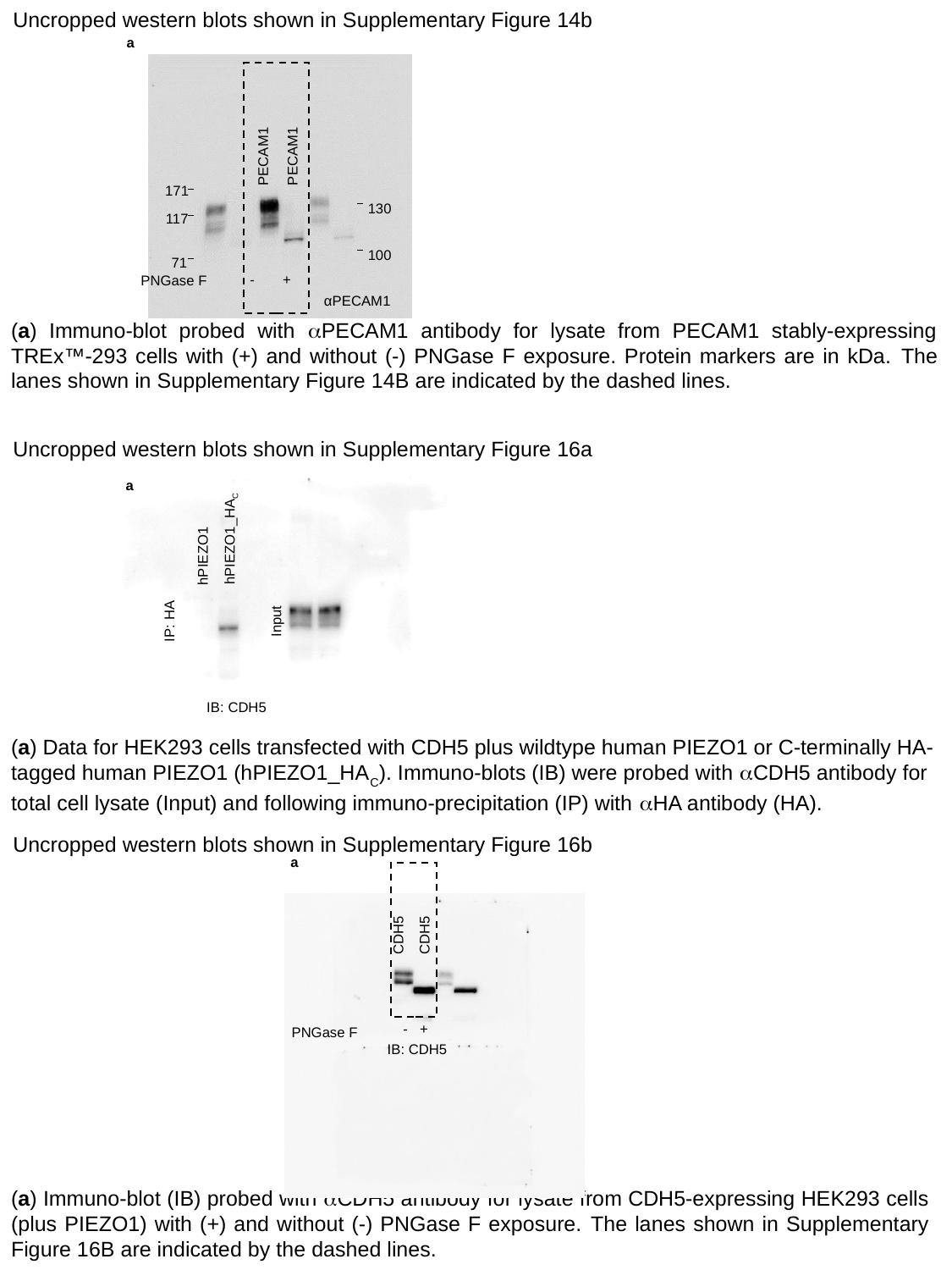

Uncropped western blots shown in Supplementary Figure 14b
a
PECAM1
PECAM1
171
130
117
100
71
- +
PNGase F
αPECAM1
(a) Immuno-blot probed with αPECAM1 antibody for lysate from PECAM1 stably-expressing TREx™-293 cells with (+) and without (-) PNGase F exposure. Protein markers are in kDa. The lanes shown in Supplementary Figure 14B are indicated by the dashed lines.
Uncropped western blots shown in Supplementary Figure 16a
a
hPIEZO1_HAC
hPIEZO1
IP: HA
Input
IB: CDH5
(a) Data for HEK293 cells transfected with CDH5 plus wildtype human PIEZO1 or C-terminally HA-tagged human PIEZO1 (hPIEZO1_HAC). Immuno-blots (IB) were probed with αCDH5 antibody for total cell lysate (Input) and following immuno-precipitation (IP) with αHA antibody (HA).
Uncropped western blots shown in Supplementary Figure 16b
a
CDH5
CDH5
- +
PNGase F
IB: CDH5
(a) Immuno-blot (IB) probed with αCDH5 antibody for lysate from CDH5-expressing HEK293 cells (plus PIEZO1) with (+) and without (-) PNGase F exposure. The lanes shown in Supplementary Figure 16B are indicated by the dashed lines.

## Slide 34
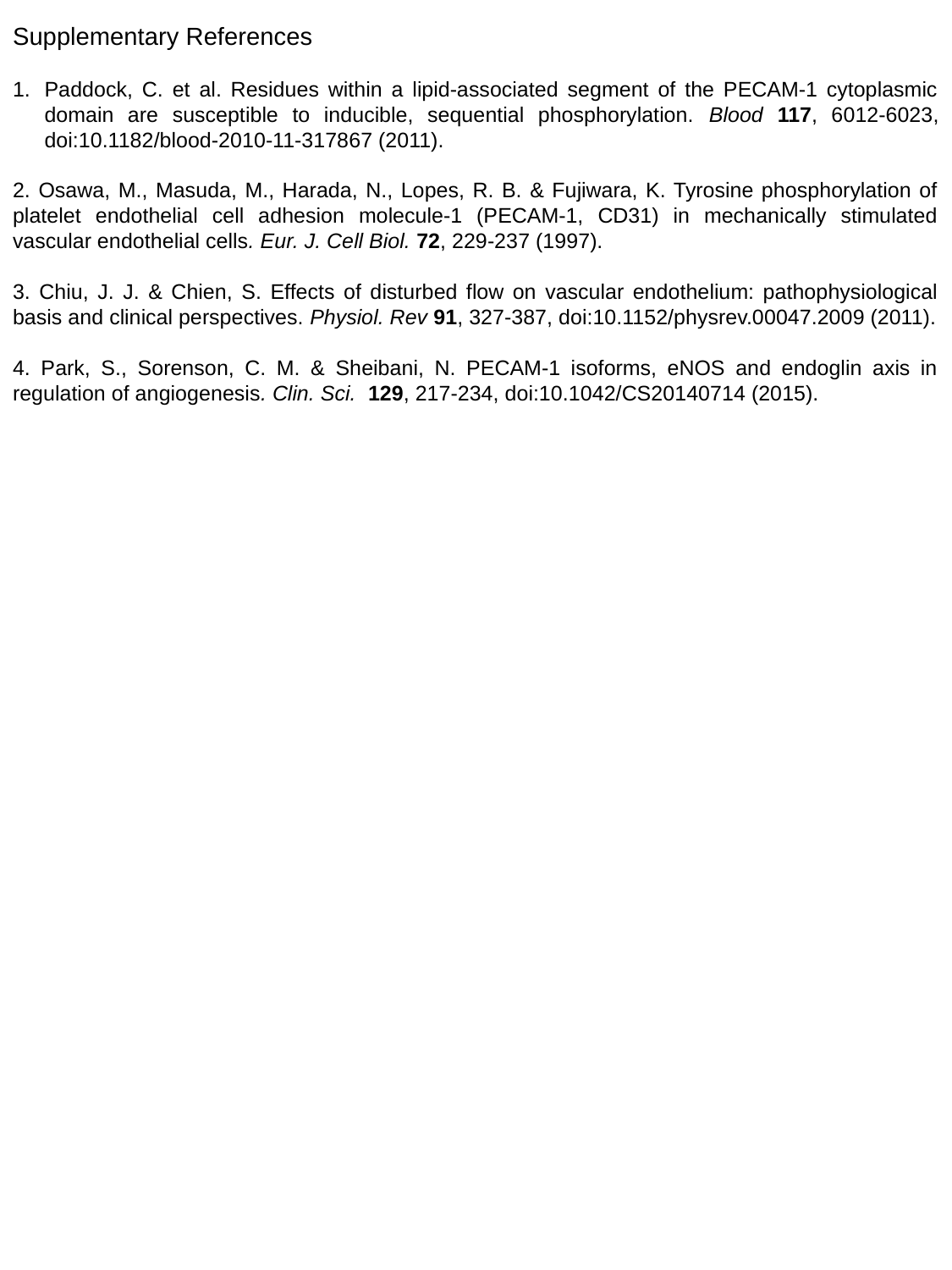

Supplementary References
Paddock, C. et al. Residues within a lipid-associated segment of the PECAM-1 cytoplasmic domain are susceptible to inducible, sequential phosphorylation. Blood 117, 6012-6023, doi:10.1182/blood-2010-11-317867 (2011).
2. Osawa, M., Masuda, M., Harada, N., Lopes, R. B. & Fujiwara, K. Tyrosine phosphorylation of platelet endothelial cell adhesion molecule-1 (PECAM-1, CD31) in mechanically stimulated vascular endothelial cells. Eur. J. Cell Biol. 72, 229-237 (1997).
3. Chiu, J. J. & Chien, S. Effects of disturbed flow on vascular endothelium: pathophysiological basis and clinical perspectives. Physiol. Rev 91, 327-387, doi:10.1152/physrev.00047.2009 (2011).
4. Park, S., Sorenson, C. M. & Sheibani, N. PECAM-1 isoforms, eNOS and endoglin axis in regulation of angiogenesis. Clin. Sci. 129, 217-234, doi:10.1042/CS20140714 (2015).
