# Supplementary material for: PIEZO1 and PECAM1 interact at cell-cell junctions and partner in endothelial force sensing
Source: Commun Biol. 2023 Apr 1;6:358. doi: 10.1038/s42003-023-04706-4 (PMC10067937; doi:10.1038/s42003-023-04706-4)
Supplement: Supplementary file 3 — Description of Additional Supplementary Data [file 42003_2023_4706_MOESM3_ESM.docx]

**Description of Additional Supplementary Files**

**File name:** Supplementary Data 1

**Description:** The source data behind the graphs in the paper.

**File name:** Supplementary Data 2

**Description:** The source data behind the graphs in the supplementary figures.

**File name:** Supplementary Data 3

**Description:** Table of primers.
