# Supplementary material for: PIEZO1 and PECAM1 interact at cell-cell junctions and partner in endothelial force sensing
Source: Commun Biol. 2023 Apr 1;6:358. doi: 10.1038/s42003-023-04706-4 (PMC10067937; doi:10.1038/s42003-023-04706-4)
Supplement: Supplementary file 6 — Supplementary Data 3 [file 42003_2023_4706_MOESM6_ESM.docx]

**SUPPLEMENTARY TABLE 1** PCR Primers

**Primers for PIEZO1-mTurquoise2 and PECAM1-SYFP2 constructs**

| **PIEZO1 and PECAM1 pcDNA6 templates** |  |
| --- | --- |
| PIEZO1 forward | TGCAGATATCCAGCACAG |
| PIEZO1 reverse | GGATCCCTCCTTCTCAC |
| PECAM1 forward | GAATTCTGCAGATATCCAGC |
| PECAM1 reverse | AAGCTTAGTTCCATCAAGG |
| **mTurquoise2 and SYFP2 insertion** |  |
| PIEZO1 BamH1 mTurqouise2/SYFP2 forward | GAGAAGGAGGGATCCATGGTGAGCAAGGGC |
| PIEZO1 BamH1 mTurqouise2/SYFP2 reverse | CTGGATATCTGCATTACTTGTACAGCTCGTCC |
| PECAM1 HindIII mTurqouise2/SYFP2 forward | CCTTGATGGAACTAAGCTTATGGTGAGCAAGGGC |
| **Linker insertion** |  |
| PIEZO1 L4 mTurquoise2/SYFP2 forward | CGTGAGAAGGAGGGATCCGCCAGCGCATCCGCTAGCGCCTCTGCCAGCGCTAGTGCCTCTGCATCCGCTTCA |
| PIEZO1 L4 mTurquoise2/SYFP2 reverse | CTTGCTCACCATGGATCCGCTAGCACTTGCGCTTGCACTGGCAGAGGCGGAGGCTGAAGCGGATGCAGA |
| PECAM1 L4 mTurquoise2/SYFP2 forward | CCCTTGATGGAACTAAGCTTGCCAGCGCATCCGCTAGCGCCTCTGCCAGCGCTAGTGCCTCTGCATCCGCTTCA |
| PECAM1 L4 mTurquoise2/SYFP2 reverse | CCTTGCTCACCATAAGCTTGCTAGCACTTGCGCTTGCACTGGCAGAGGCGGAGGCTGAAGCGGATGCAGA |

**Primers for PECAM1-SYFP2 mutagenesis**

| C622A forward | CATTGCGGCCAAACGTTATTTTCTGAGG |
| --- | --- |
| C622A reverse | CCTCAGAAAATAACGTTTGGCCGCAATG |
| Y663F forward | GCTAACAGTCATTTCGGTCACAATGAC |
| Y663F reverse | GTCATTGTGACCGAAATGACTGTTAGC |
| Y690F forward | CTCAGACGTGCAGTTCACGGAAGTTCAAG |
| Y690F reverse | CTTGAACTTC CGTGAACTGCACGTCTGAG |
| S700F forward | GTCCTCAGCTGAGTTTCACAAAGATCTAG |
| S700F reverse | CTAGATCTTTGTGAAACTCAGCTGAGGAC |
| Y713F forward | CACAGAGACAGTGTTCAGTGAAGTCCGG |
| Y713F reverse | CCGGACTTCACTGAACACTGTCTCTGTG |

**Primers for PECAM1-ext-SYFP2**

| Ext forward | GGACTCAGATCTCGAGCTCAACAAGAAAACTCTTTCACAATC |
| --- | --- |
| Ext reverse | GACTGCAGAATTCGAAGCTTTCTTCCATGGGGCAAGAAT |
| PIN-G forward | GCTTCGAATTCTGCAGTCGACGAACAAAAACTCATC |
| PIN-G reverse | CTGTACAAGTCCGGACTCAGATCTCGAGCTCAA |

**Primers for VGFR2-SYFP2**

| CT deletion forward | CTCCTGTGGATTCCTACCAGTACGGCACCAC |
| --- | --- |
| CT deletion reverse | GTGGTGCCGTACTGGTAGGAATCCACAGGAG |
| I297V mutagenesis forward | CTTAACTATAGATGGTGTAACCCGGAGTGACC |
| I297V mutagenesis reverse | GGTCACTCCGGGTTACACCATCTATAGTTAAG |
| Q472H mutagenesis forward | CAACGAGCCCAGCCATGCTGTCTCAGTGAC |
| Q472H mutagenesis reverse | GTCACTGAGACAGCATGGCTGGGCTCGTTG |
| Linker forward | CCACTAGTCCAGTGTGGTGGAATTCACCGGTGCCAGCGCATCCGCTTCTGCCTCCGCAAGCGCTAGTGCCTCTGC |
| Linker reverse | GTTTAAACGGGCCCTCTAGACTCGAGCCGCGGGAGGCTGAAGCGGATGCAGAGGCACTAGCGCTTGCG |
| SYFP2 forward | TAATGGTACCGCCACCATGGTGAGC |
| SYFP2 reverse | TATTACCGGTCTTGTACAGCTCGTCCATGC |
| Overlapping VGFR2 forward | GATCCGCCACCATGCAGAGC |
| Overlapping VGFR2 reverse | GCCCTCTAGATTAAACAGGAGGAGAGCTCAGTGTG |
| pcDNA4/TO sYPF2 forward | CTCCTCCTGTTTAATCTAGAGGGCCCGTTTAAAC |
| pcDNA4/TO sYPF2 reverse | CTCTGCATGGTGGCGGATCCG |
| Assemble VGFR2 forward | GATCCGCCACCATGCAGAGC |
| Assemble VGFR2 reverse | GCCCTCTAGATTAAACAGGAGGAGAGCTCAGTGTG |
| Assemble vector forward | CTCCTCCTGTTTAATCTAGAGGGCCCGTTTAAAC |
| Assemble vector reverse | CTCTGCATGGTGGCGGATCCG |

**Primers for HA-PIEZO1**

| PIEZO1 PCR forward | CGGAAAGGAGCGGCAGCCATCG |
| --- | --- |
| PIEZO1 PCR reverse | TAATGCGGCCGCTTACTCCTTCTCACGAGTCCACTTG |
| Universal forward primer | GGTCTACCTGCTCTTCCTGCTG |
| Full length PIEZO1 | CGTAATCTGGAACATCGTATGGGTAAGCTGATGCCTCCTTCTCACGAGTCCACTTG |
| L2471 reverse | CGTAATCTGGAACATCGTATGGGTAAGCTGATGCCAGCTCCTCGAACATAATGGAG |
| G2174 reverse | CGTAATCTGGAACATCGTATGGGTATGCGCCGTACTTGACGATCTTCTTC |
| I2089 reverse | CGTAATCTGGAACATCGTATGGGTATGCGATGCGGGTGGGGTAGC |
| S1591 reverse | CGTAATCTGGAACATCGTATGGGTATGCGGACACGGTGCTTGGGG |
| A1128 reverse | CGTAATCTGGAACATCGTATGGGTATGCAGCCATGCGCTGCCAC |
| Insertion forward | CCCATACGATGTTCCAGATTACGCTTAGGCGACTCTAGATCATAATCAGCCATACC |
| Insertion reverse | CAGCAGGAAGAGCAGGTAGACC |

**Primers for Mouse PIEZO1**

| PIEZO1 Forward primer | GTAACAACTCCGCCCCATTG |
| --- | --- |
| PIEZO1 Reverse primer | GCTTCTACTCCCTCTCACGTGTC |
| pcDNA™4/TO Forward | GACACGTGAGAGGGAGTAGAAGCCGCTGATCAGCCTCGACTG |
| pcDNA™4/TO Reverse | CAATGGGGCGGAGTTGTTAC |

**Primers for PIEZO1-GFP**

| pGNP5-1-F | TATAGGGAGACCCAAGCTGGTTAACGCCACCATGGAGCCGCACGTGCTC |
| --- | --- |
| pGNP5-1-R | GAGGCTGATCAGCGGTTTAAACTTAAGTCACTTGTACAGCTCATCCA |
