# Supplementary material for: PIEZO1 and PECAM1 interact at cell-cell junctions and partner in endothelial force sensing
Source: Commun Biol. 2023 Apr 1;6:358. doi: 10.1038/s42003-023-04706-4 (PMC10067937; doi:10.1038/s42003-023-04706-4)
Supplement: Supplementary file 7 — Reporting summary [file 42003_2023_4706_MOESM7_ESM.pdf]

Corresponding author(s): Eulashini Chuntharpursat-Bon and David J. Beech

Last updated by author(s): 2023/03/01

Reporting Summary

Nature Portfolio wishes to improve the reproducibility of the work that we publish. This form provides structure for consistency and transparency in reporting. For further information on Nature Portfolio policies, see our Editorial Policies and the Editorial Policy Checklist.

Statistics

For all statistical analyses, confirm that the following items are present in the figure legend, table legend, main text, or Methods section.

n/a	Confirmed	
☐	☒	The exact sample size (*n*) for each experimental group/condition, given as a discrete number and unit of measurement
☐	☒	A statement on whether measurements were taken from distinct samples or whether the same sample was measured repeatedly
☐	☒	The statistical test(s) used AND whether they are one- or two-sided<br>*Only common tests should be described solely by name; describe more complex techniques in the Methods section.*
☐	☒	A description of all covariates tested
☐	☒	A description of any assumptions or corrections, such as tests of normality and adjustment for multiple comparisons
☐	☒	A full description of the statistical parameters including central tendency (e.g. means) or other basic estimates (e.g. regression coefficient) AND variation (e.g. standard deviation) or associated estimates of uncertainty (e.g. confidence intervals)
☐	☒	For null hypothesis testing, the test statistic (e.g. *F*, *t*, *r*) with confidence intervals, effect sizes, degrees of freedom and *P* value noted<br>*Give P values as exact values whenever suitable.*
☒	☐	For Bayesian analysis, information on the choice of priors and Markov chain Monte Carlo settings
☒	☐	For hierarchical and complex designs, identification of the appropriate level for tests and full reporting of outcomes
☒	☐	Estimates of effect sizes (e.g. Cohen's *d*, Pearson's *r*), indicating how they were calculated

Our web collection on statistics for biologists contains articles on many of the points above.

Software and code

Policy information about availability of computer code

Data collection	Zen black (Zeiss)<br>Digidata 1550B and pClamp 10.6 software (Molecular Devices, USA)<br>SPCImage 5.6 (Becker and Hickl)<br>SoftMax Pro 5.4.5 software (Molecular Devices)<br>STEDYCON (Arberrior)
Data analysis	Fiji (ImageJ)<br>OriginPro 2018 (OriginLab Corporation, USA)<br>SPCImage 5.6 (Becker and Hickl)

For manuscripts utilizing custom algorithms or software that are central to the research but not yet described in published literature, software must be made available to editors and reviewers. We strongly encourage code deposition in a community repository (e.g. GitHub). See the Nature Portfolio guidelines for submitting code & software for further information.

Data

Policy information about availability of data

All manuscripts must include a data availability statement. This statement should provide the following information, where applicable:

- Accession codes, unique identifiers, or web links for publicly available datasets
- A description of any restrictions on data availability
- For clinical datasets or third party data, please ensure that the statement adheres to our policy

Source data are available upon request.

Human research participants

Policy information about studies involving human research participants and Sex and Gender in Research.

Reporting on sex and gender	*Use the terms sex (biological attribute) and gender (shaped by social and cultural circumstances) carefully in order to avoid confusing both terms. Indicate if findings apply to only one sex or gender; describe whether sex and gender were considered in study design whether sex and/or gender was determined based on self-reporting or assigned and methods used. Provide in the source data disaggregated sex and gender data where this information has been collected, and consent has been obtained for sharing of individual-level data; provide overall numbers in this Reporting Summary. Please state if this information has not been collected. Report sex- and gender-based analyses where performed, justify reasons for lack of sex- and gender-based analysis.*
Population characteristics	*Describe the covariate-relevant population characteristics of the human research participants (e.g. age, genotypic information, past and current diagnosis and treatment categories). If you filled out the behavioural & social sciences study design questions and have nothing to add here, write "See above."*
Recruitment	*Describe how participants were recruited. Outline any potential self-selection bias or other biases that may be present and how these are likely to impact results.*
Ethics oversight	*Identify the organization(s) that approved the study protocol.*

Note that full information on the approval of the study protocol must also be provided in the manuscript.

Field-specific reporting

Please select the one below that is the best fit for your research. If you are not sure, read the appropriate sections before making your selection.

☒ Life sciences ☐ Behavioural & social sciences ☐ Ecological, evolutionary & environmental sciences

For a reference copy of the document with all sections, see nature.com/documents/nr-reporting-summary-flat.pdf

Life sciences study design

All studies must disclose on these points even when the disclosure is negative.

Sample size	The study was aimed at deciphering a biological mechanism. In the absence of prior knowledge of this mechanism, power calculations were not considered to be applicable.<br>We selected numbers of independent repeats of experiments base on prior experience of studies of this type. This statement is included in the Methods section under Data Analysis.
Data exclusions	No data were excluded; outliers are shown on box plots
Replication	In the Methods “Data Analysis”, we state: “For quantitative data, 3 independent repeats (n) were performed with a minimum of 3 technical repeats each.”<br>The number of independent experiments is separately described in each figure legend.
Randomization	Samples were allocated randomly to groups.<br>This was not a clinical study.
Blinding	In the Methods, we state: “The person performing the patch measurements was blinded to the constructs that had been transfected into the cells.

Reporting for specific materials, systems and methods

We require information from authors about some types of materials, experimental systems and methods used in many studies. Here, indicate whether each material, system or method listed is relevant to your study. If you are not sure if a list item applies to your research, read the appropriate section before selecting a response.

Materials & experimental systems

n/a	Involved in the study
☐	☒ Antibodies
☐	☒ Eukaryotic cell lines
☒	☐ Palaeontology and archaeology
☐	☒ Animals and other organisms
☒	☐ Clinical data
☒	☐ Dual use research of concern

Methods

n/a	Involved in the study
☒	☐ ChIP-seq
☒	☐ Flow cytometry
☒	☐ MRI-based neuroimaging

Antibodies

Antibodies used

Anti-GFP Abcam Cat#ab1218
Anti-GST GE Healthcare Cat#27457701
Anti-mouse 647 Thermofisher Cat#A21240
Anti-mouse Alexa 594 Jackson Immuno Research Cat#115-585-146
Anti-mouse STAR red Aberrior Cat#52283
Anti-Piezo1 Proteintech Cat#15939-1-AP
Anti-Piezo1 BEEC4 (custom designed antibody) Cambridge Biosciences
Anti-rabbit 488 Jackson ImmunoResearch Cat#711-545-152
Anti-rabbit 580 Aberrior Cat#41367
Anti-β-actin Santa Cruz Cat#sc47778
Goat anti-rabbit 647 Invitrogen Cat#A21246
Goat anti-rat 488 Invitrogen Cat#A11006
Horseradish peroxidase donkey anti-goat Jackson ImmunoResearch Cat#705-035-003
Horseradish peroxidase donkey anti-mouse Jackson ImmunoResearch Cat#715-035-150
Horseradish peroxidase donkey anti-rat Jackson ImmunoResearch Cat#712-035-150
Horseradish peroxidase goat anti-mouse Jackson ImmunoResearch Cat#115-035-003
Horseradish peroxidase rabbit anti-goat Jackson ImmunoResearch Cat#305-035-003
Mouse anti-CD31 DAKO Clone JC70A
Mouse anti-halo Promega Cat#G9211
Mouse anti-VE-Cadherin R&D Systems Cat#MAB9381
Rabbit anti-HA Cell Signaling Technology Cat#3724
Rabbit anti-HA Cell Signalling Cat#mAB3724
Rabbit anti-VE-cadherin Abcam Cat#AB33168
Rat anti-HA Roche Clone 3F10
Rat anti-mouse CD31 BD Pharmingen™ Cat#550274

Validation

Negative controls are described for each primary antibody in the figure legends

Eukaryotic cell lines

Policy information about cell lines and Sex and Gender in Research

Cell line source(s)

COS-7 ATCC Cat#CRL-1651
Griptite™ 293 MSR Thermofisher Cat#11668019
HEK293 ATCC Cat#CRL-1573
Human CD31 T-REx-293 This paper
Human Piezo1 T-REx-293 Rode et. al., 2017
HUVEC PromoCell Cat#C-12203
Mouse Piezo1 T-Rex-293 This paper

Authentication

None of the cell lines was authenticated

Mycoplasma contamination

All cell lines tested negative for mycoplasm contamination

Commonly misidentified lines
(See ICLAC register)

HEK 293 cells were used for overexpression studies. They are commonly used in the field .

Animals and other research organisms

Policy information about studies involving animals; ARRIVE guidelines recommended for reporting animal research, and Sex and Gender in Research

Laboratory animals

C57BL/6 Piezo1 HA This paper
C57BL/6J wild type Charles River

	Male,<br>10-14 weeks old
Wild animals	No wild animals were studied.
Reporting on sex	Only male mice were studied, to avoid variability in female mice arising due to the estrus cycle and because determination of differences in the mechanism between sexes was not an objective.
Field-collected samples	No field-collected samples were used. Laboratory mice were used. We state that: All animal use was authorized by the University of Leeds Animal Ethics Committee and The Home Office, UK. Animals were maintained in GM500 individually ventilated cages (Animal Care Systems) at 21 °C, 50–70% humidity, light/dark cycle 12/12 h on chow diet ad libitum and bedding of Pure'o Cell (Special Diet Services, Datesand Ltd, Manchester, UK). Genotypes were determined using real-time PCR with specific probes designed for each gene (Transnetyx, Cordova, TN).
Ethics oversight	All animal use was authorized by the University of Leeds Animal Ethics Committee and The Home Office, UK.

Note that full information on the approval of the study protocol must also be provided in the manuscript.
